# Characterization of Inflammatory Bowel Disease Heterogeneity Using Serum Proteomics: A Multicenter Study

**DOI:** 10.1093/ecco-jcc/jjae169

**Published:** 2024-11-04

**Authors:** Benita Salomon, Padhmanand Sudhakar, Daniel Bergemalm, Erik Andersson, Olle Grännö, Marie Carlson, Charlotte R H Hedin, Johan D Söderholm, Lena Öhman, the BIO IBD Consortium, the COLLIBRI Consortium, Ryan C Ungaro, Konrad Aden, Geert D’Haens, Mark S Silverberg, Sven Almer, Francesca Bresso, Adam Carstens, Mauro D’Amato, Carl Eriksson, Henrik Hjortswang, Åsa V Keita, Maria Ling Lundström, Maria K Magnusson, Jóhann P Hreinsson, Hans Strid, Carl Mårten Lindqvist, Robert Kruse, Dirk Repsilber, Bram Verstockt, Séverine Vermeire, Jonas Halfvarson

**Affiliations:** Faculty of Medicine and Health, School of Medical Sciences, Örebro University, Örebro, Sweden; Department of Chronic Diseases and Metabolism, Translational Research Center for Gastrointestinal Disorders (TARGID), KU Leuven, Leuven, Belgium; Department of Biotechnology, Kumaraguru College of Technology, Coimbatore, Tamil Nadu, India; Faculty of Medicine and Health, Department of Gastroenterology, Örebro University, Örebro, Sweden; Faculty of Medicine and Health, Department of Gastroenterology, Örebro University, Örebro, Sweden; Faculty of Medicine and Health, Department of Laboratory Medicine, Clinical Microbiology, Örebro University, Örebro, Sweden; Department of Medical Sciences, Gastroenterology Research Group, Uppsala University, Uppsala, Sweden; Department of Medicine Solna, Karolinska Institute, Stockholm, Sweden; Department of Gastroenterology, Dermatovenereology, and Rheumatology, Centre for Digestive Health, Karolinska University Hospital, Stockholm, Sweden; Department of Surgery, Linköping University, Linköping, Sweden; Department of Biomedical and Clinical Sciences, Linköping University, Linköping, Sweden; Department of Microbiology and Immunology, Institute of Biomedicine, Sahlgrenska Academy, University of Gothenburg, Göteborg, Sweden; Faculty of Medicine and Health, School of Medical Sciences, Örebro University, Örebro, Sweden; Faculty of Medicine and Health, School of Medical Sciences, Örebro University, Örebro, Sweden; Faculty of Medicine and Health, Inflammatory Response and Infection Susceptibility Centre (iRiSC), Örebro University, Örebro, Sweden; Faculty of Medicine and Health, Department of Clinical Research Laboratory, Örebro University, Örebro, Sweden; Faculty of Medicine and Health, School of Medical Sciences, Örebro University, Örebro, Sweden; Department of Chronic Diseases and Metabolism, Translational Research Center for Gastrointestinal Disorders (TARGID), KU Leuven, Leuven, Belgium; Department of Gastroenterology and Hepatology, KU Leuven, University Hospitals Leuven, Leuven, Belgium; Department of Chronic Diseases and Metabolism, Translational Research Center for Gastrointestinal Disorders (TARGID), KU Leuven, Leuven, Belgium; Faculty of Medicine and Health, Department of Gastroenterology, Örebro University, Örebro, Sweden

**Keywords:** Crohn’s disease, ulcerative colitis, disease location, serum proteins, Montreal classification, biomarkers

## Abstract

**Background:**

Recent genetic and transcriptomic data highlight the need for improved molecular characterization of inflammatory bowel disease (IBD). Proteomics may advance the delineation of IBD phenotypes since it accounts for post-transcriptional modifications.

**Aims:**

We aimed to assess the IBD spectrum based on inflammatory serum proteins and identify discriminative patterns of underlying biological subtypes across multiple European cohorts.

**Methods:**

Using proximity extension methodology, we measured 86 inflammation-related serum proteins in 1551 IBD patients and 312 healthy controls (HC). We screened for proteins exhibiting significantly different levels among IBD subtypes and between IBD and HC. Classification models for differentiating between Crohn’s disease (CD) and ulcerative colitis (UC) were employed to explore the IBD spectrum based on estimated probability scores.

**Results:**

Levels of multiple proteins, such as interleukin-17A, matrix metalloproteinase-10, and fibroblast growth factor-19, differed (fold-change >1.2; false discovery rate <0.05) between ileal versus colonic IBD. Using multivariable models, a protein signature reflecting the IBD spectrum was identified, positioning colonic CD between UC and ileal CD, which were at opposite ends of the spectrum. Based on area under the curve (AUC) estimates, classification models more accurately differentiated UC from ileal CD (median AUCs > 0.73) than colonic CD (median AUCs < 0.62). Models differentiating colonic CD from ileal CD demonstrated intermediate performance (median AUCs: 0.67–0.69).

**Conclusions:**

Our findings in serum proteins support the presence of a continuous IBD spectrum rather than a clear separation of CD and UC. Within the spectrum, disease location may reflect a more similar disease than CD versus UC, as colonic CD resembled UC more closely than ileal CD.

## Graphical Abstract

**Graphical Abstract:**
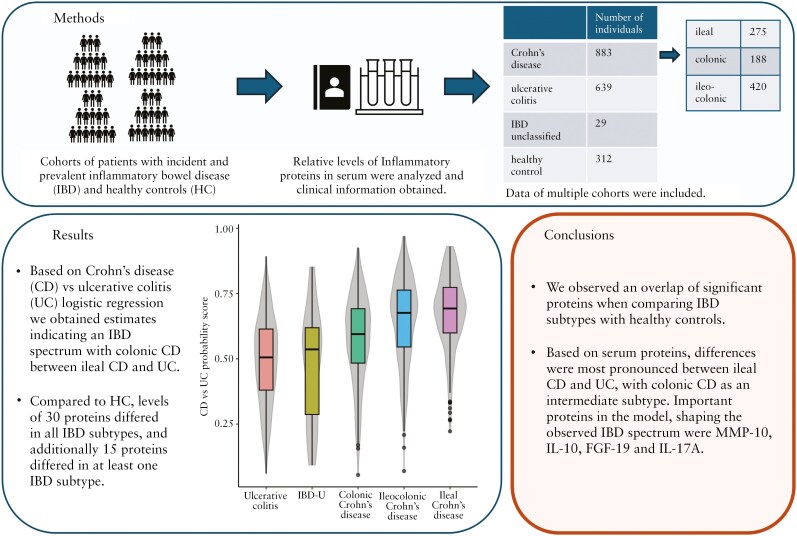

## 1. Introduction

Inflammatory bowel disease (IBD) is an umbrella term for chronic inflammatory disorders of the gastrointestinal tract, with Crohn’s disease (CD) and ulcerative colitis (UC) as the 2 main phenotypes, and with IBD-unclassified (IBD-U) as an entity for patients with features of colonic IBD but in whom no further distinction can be made.^1,2^ In addition, there is no single CD or UC phenotype, as both entities are characterized by a broad heterogeneity with differences in clinical presentation.^3^ To account for this heterogeneity, classifications have been developed to stratify patients. The original Vienna classification^4^ was followed by the Montreal classification, which stratifies patients by location, behavior, and age at diagnosis in CD, as well as by extent of inflammation in UC.^5^ However, current classifications do not capture the heterogeneity faced in daily clinical practice and, therefore, need to be further refined.^6^

Despite clinical and potentially underlying pathophysiological differences within each diagnostic entity, all CD and UC patients are combined in randomized clinical trials per disease entity, not taking the heterogeneity and different subphenotypes into account.^7,8^ This lack of adequate stratification may contribute to challenges in optimized therapeutic strategies. Consequently, improved molecular profiling of IBD phenotypes is needed. Some studies support the separation in subphenotypes, where genetic and transcriptomic data suggest an IBD continuum with ileal CD and UC at both extremes.^9–12^ By calculating genetic risk scores, Cleynen et al. introduced this continuum concept with colonic CD in between UC and ileal CD.^11^ Based on accumulating data findings in IBD, an emerging perspective suggests the need to redefine the traditional classification.^6,9^ However, a disease classification based on only single nucleotide polymorphisms does not account for post-transcriptional modifications or environmental and lifestyle influences. Analyses of serum proteins could provide additional insights into the underlying inflammatory mechanisms and help delineate various IBD subphenotypes. Therefore, the aim of this study was to analyze inflammatory serum proteins from multiple European cohorts of patients with different disease duration, activity, and treatment exposures, to delineate the heterogeneity of the spectrum of IBD and identify discriminative patterns linked to underlying molecular subtypes.

## 2. Methods

### 2.1. Study design

The Collaborative IBD Biomarker Research Initiative (COLLIBRI) aims to enable IBD biomarker research with larger sample sizes by combining existing datasets of multiple centers. Here, we conducted a cross-sectional study to investigate molecular subgroup-specific patterns from existing datasets derived from patients with IBD and healthy controls (HC).

### 2.2. Patient cohort

Cohorts within the consortium with pre-existing protein data generated using the Olink inflammation panel (Olink Proteomics) were included.^13^ IBD diagnosis was based on internationally accepted criteria, following thorough clinical, microbiological, endoscopic, histological, and radiological evaluation in all cohorts.^14^ Phenotypes were defined using the currently established Montreal classification.^5^ We included data of patients and controls from several different cohorts, including 2 cohorts of patients with prevalent IBD at Örebro University Hospital, Sweden (*n* = 446), the Swedish Inception Cohort (*n* = 320), and the Swedish cohort of patients starting biological treatment (*n* = 245), comprising samples from 7 centers in Sweden, and also from KU Leuven, Belgium (*n* = 852). Further details about these cohorts are available in Supplementary Table 1.

### 2.3. Ethical approval

The study was approved by the regional ethics committee/The Swedish Ethical Review Authority (Dnr 2010/313, 2011-01-12; amendments Dnr 2010/313/1, Dnr 2010/313/2, Dnr 2010/313/3, Dnr 2010/313/4 (Dnr 2020-03547), Dnr 2022-03231-02, Dnr 2023-01901-02, 2023-02919-02, Dnr 2024-04489-02) and by the Ethical Committee of the University Hospitals of Leuven (B322201213950/S53684). Informed consent was obtained from all participants at inclusion in the study.

### 2.4. Protein analysis

Serum samples were analyzed using the Proseek Multiplex Inflammation I Probe kit 96 × 96 (Olink Bioscience, Uppsala, Sweden), including 92 inflammation-related proteins as previously described in detail.^13^ Data normalization and standardization were performed using the Olink Wizard for GenEx (Multid Analysis, Sweden). In these steps, the Ct values of the qPCR were transformed into the arbitrary unit, Normalized Protein eXpression (NPX), which represents the relative protein levels on the log2 scale.^15^

### 2.5. Data preprocessing

The data used in this study were generated prior to this project and were obtained from different cohorts and runs. Therefore, adjustments for batch effects were necessary, and these adjustments are described in the Supplementary Material. Samples that did not pass the quality check based on Olink’s quality criteria were excluded.^15^ The proteins Cluster of Differentiation 8a (CD8A) and brain-derived neurotrophic factor were not available in all batches due to changes in the Olink Inflammation panel over time and were therefore omitted from the analyses. Furthermore, tumor necrosis factor (TNF) and interferon-gamma (IFN-gamma) assays were changed over time, and these 2 proteins were also excluded. In addition, 3 proteins with >90% values below the limit of detection were excluded: interleukin (IL)-1 alpha, IL-22 receptor alpha 1 (IL-22RA1), and IL-33.

### 2.6. Data analysis

Data analyses were performed using R,^16^ version 4.05, and relevant packages as listed in the Supplementary Material. Continuous clinical data and data on an ordinal scale are presented as median and range or interquartile range (IQR), and differences were tested with the Mann–Whitney *U*-test, 2-sided, with a significance level of 0.05 and nominal *p*-values reported. Categorical data are presented as frequencies and were compared using the chi-square test.

Principal component analysis (PCA) and Uniform Manifold Approximation and Projection (UMAP) were used to identify potential subgroups of patients with IBD based on the results of the inflammatory serum proteome.

Univariate pairwise comparisons of subgroups were performed using Welsh *t*-tests, followed by the calculation of Benjamini–Hochberg’s false discovery rate (FDR) values to control for multiple testing.^17^ The log2-fold-change values for all pairwise group comparisons were calculated. A threshold of FDR < 0.05 and an absolute fold-change threshold of 1.2, or log2-fold-change of log2(1.2), was chosen, similar to the approach previously described.^18^ For robustness, we repeated these analyses, adjusting for age, sex, and disease duration, as described in the Supplementary Material. Only individuals without previous IBD-related surgery were included in these adjusted analyses to account for potential differences due to surgery.

In line with the adopted strategy for genetic risk scores by Cleynen et al.,^11^ we computed CD versus UC probability scores using logistic regression models. More specifically, we employed combined Smoothly Clipped Absolute Deviation (SCAD) and L2 (Ridge) penalized logistic regression (PLR)^19^ using the ncvreg package.^20^ The parameter alpha, which determines the relative contribution of SCAD and L2 penalty in the penalization term, was set to 0.1. The maximal number of proteins (variables) included in the model was set to 28. We used nested cross-validation for parameter optimization and to estimate predictive performance. The regularization parameter lambda was optimized through an inner 5-fold cross-validation. We used outer leave-one-out cross-validation, estimating the probability of a specific diagnosis for each left-out sample within the range of 0–1. Ten models were fitted and the average probability estimate was used for each left-out sample. The median CD versus UC predicted probability scores based on protein models (referred to as CD vs. UC probability scores) were calculated for each subgroup. Differences between mean probability scores were compared using 1-way analysis of variance followed by Tukey’s Honestly Significant Difference test and their adjusted confidence intervals were calculated. Protein contributions in the models were visualized by displaying model coefficients as boxplots. Thereby, the absolute coefficients indicate importance in the model. While positive coefficients contribute to a score closer to 1 (CD), negative coefficients contribute to a score closer to 0 (UC). The coefficients were estimated by fitting the models with all data in 1000 repetitions. Due to the randomness of the inner cross-validation and the related choice of lambda, the coefficients differed between models. We also estimated the performance of these scores to classify the subgroups, using the area under the receiver-operating-characteristic curve (AUC). As an additional approach to investigate possible influences of subgroup-specific medications and robustness, a CD versus UC model was fitted without including data from the Swedish inception cohort (SIC IBD). This model was then applied to the treatment-naïve patients in the SIC IBD to control for potential treatment effects.

Furthermore, probability scores were estimated using a Random Forest (RF) model using the R-package ranger to explore potential method dependency.^21^ We also investigated the subtype-specific differences using partial least square analysis as described in the Supplementary Material.

We employed SCAD/Ridge-PLR, RF, least absolute shrinkage, and selection operator (Lasso)-PLR,^22^ and regularized support vector machines with a radial kernel^23^ to examine the capacity of different types of classification models to discriminate IBD subgroups based on protein signatures. Repeated outer 5-fold cross-validation was applied and the AUC was used to assess model performance. We implemented repeated random down-sampling within the cross-validation procedure to prevent differences due to unequal group sizes.

## 3. Results

### 3.1. Clinical markers and patient characteristics

In total, data from protein analyses were obtained from 1551 adult patients (≥18 years) with IBD (CD, *n* = 883; UC, *n* = 639 and IBD-U, *n* = 29) and 312 HC (Table 1). The age distribution across subgroups (CD, UC, IBD-U, and HC) exhibited no statistical difference (*p* = 0.08). However, there was a predominance of male patients in the UC group compared to the CD group (*p* < 0.01). Detailed information about available C-reactive protein (CRP) and fecal calprotectin (FCP) levels across subgroups of individuals is shown in Supplementary Tables 5 and 6. Among individuals with available CRP (n = 1285), the median CRP was numerically highest in ileocolonic CD (7.3 mg/L), followed by ileal CD (4.7 mg/L), colonic CD (4.1 mg/L), and UC (2.7 mg/L), and lowest in the HC group (0.77 mg/L). Similarly, median FCP levels (*n* = 517) were highest in colonic CD (716.0 µg/g), followed by UC (579.0 µg/g), ileocolonic CD (487.5 µg/g), ileal CD (298.0 µg/g), and lowest in the HC (10.3 µg/g).

**Table 1.** Demographics and clinical characteristics of patients with inflammatory bowel disease and healthy controls.

		Crohn’s disease, *N* = 883	Ulcerative colitis, *N* = 639	IBD-unclassified, *N* = 29	Healthy controls, *N* = 312
Sex, *n* (%)	Male	416 (47.1)	357 (55.9)	19 (65.5)	158 (50.6)
Median (IQR) Age, years		37 (26–51)	38 (28–53)	33 (28–47)	40 (27–55)
Smoking, *n* (%)	Never	365 (41.3)	275 (43.0)	12 (41.4)	34 (10.9)
	Current	161 (18.2)	50 (7.8)	1 (3.4)	3 (1.0)
	Previous	147 (16.6)	130 (20.3)	11 (37.9)	8 (2.6)
	Missing	210 (23.8)	184 (28.8)	5 (17.2)	267 (85.6)
Median (IQR) disease duration, years		7 (0–20)	4 (0–13)	0 (0–0)	
CD location, *n* (%)	Ileal (L1)	275 (31.1)			
	Colonic (L2)	188 (21.1)			
	Ileocolonic (L3)	420 (47.6)			
	Upper GI (L4)	29 (3.3)			
CD behavior, *n* (%)	Non-stricturing, non-penetrating (B1)	371 (42.0)			
	Stricturing (B2)	273 (30.9)			
	Penetrating (B3)	239 (27.1)			
	Perianal disease, *n* (%) ^*^	184 (20.8)			
UC extent, *n* (%)	Proctitis (E1)		105 (16.4)		
	Left-sided colitis (E2)		220 (34.4)		
	Extensive colitis (E3)		314 (49.1)		
Previous IBD	Yes	263 (29.8)	33 (5.2)	0 (0.0)	
surgery, *n* (%) ^**^					

Abbreviations: CD, Crohn’s disease; GI, gastrointestinal tract; IBD, inflammatory bowel disease; IBD-U, IBD-unclassified; IQR, interquartile range; UC, ulcerative colitis.

^*^Missing information for 10 patients with CD.

^**^Missing information for 126 patients with CD, 115 patients with UC, 4 patients with IBD-unclassified.

### 3.2. Overall protein profiles by diagnosis

With unsupervised multivariable analyses, PCA score plots were generated to detect potential subgroups of patients with IBD whose inflammatory protein profile differed from other patients (Supplementary Figure 1). We could not identify any subgroup-related clusters of individuals among patients with IBD (*n* = 1551). Healthy controls (*n* = 312) largely overlapped with the patients with IBD, but PC1 scores differed between HC and IBD (*p* < 0.0001). No clear pattern related to the overall inflammatory serum proteome was observed between different locations of CD in the unsupervised PCA. However, the spatial separation between patients with ulcerative proctitis (E1) and HC was less pronounced compared to the delineation between patients with extensive colitis (E3) and HC, with left-sided colitis (E2) intermediate between E1 and E3, based on Kruskal–Wallis test and Dunn’s post hoc test (Supplementary Figure 2). A loading plot showing the top 20 proteins with the highest loadings is displayed in Supplementary Figure 3. Similar results were obtained using UMAP (Supplementary Figure 4).

### 3.3. Individual protein levels by diagnosis and phenotypes

**Figure 1.**
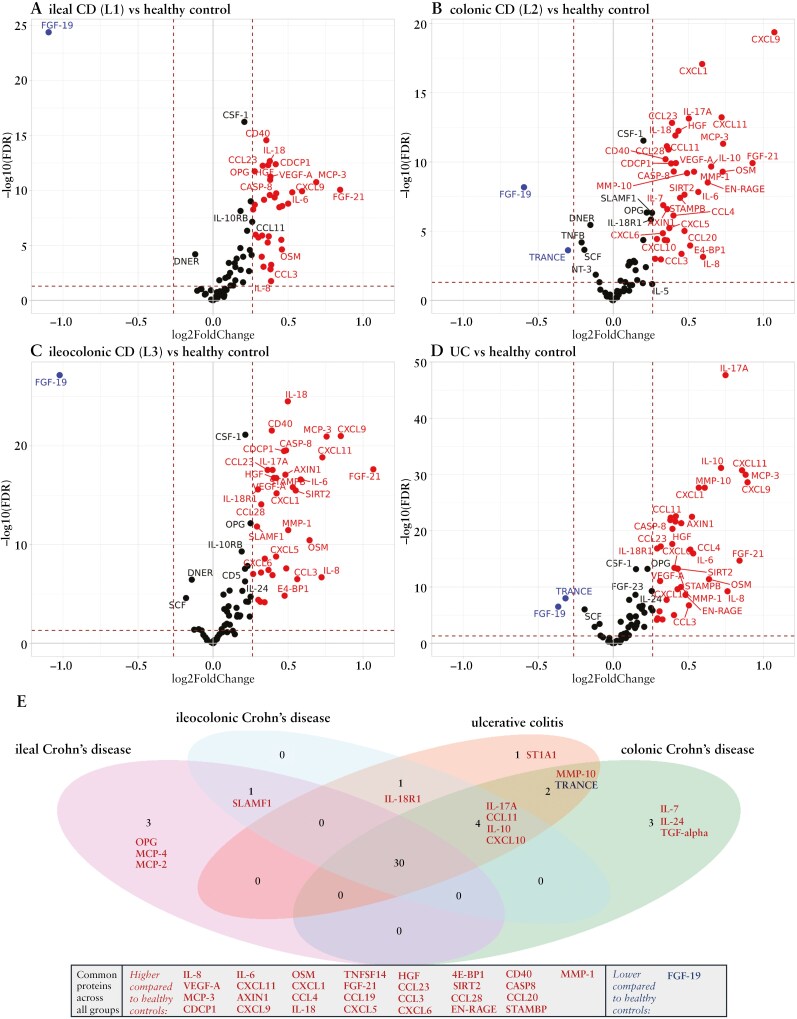
Volcano plots of differential regulated inflammatory proteins in patients with ulcerative colitis (UC) and phenotypes of Crohn’s disease (CD) (*N* = 1551) compared to healthy controls (HC) (*N* = 312). Differentially regulated proteins are annotated and were selected based on fold-change >1.2 (equal to ~0.26 on a log2 scale) and significance (false discovery rate [FDR] < 0.05). Proteins with higher relative levels in CD subgroups or UC compared to HC are to the right, proteins with lower relative levels in CD or UC compared to HC are to the left. (A) ileal CD versus HC; (B) colonic CD versus HC; (C) ileocolonic CD versus HC; and (D) UC versus HC. (E) The Venn diagram visualizes proteins with higher estimates and lower estimates in patients with UC and with and ileal-, ileocolonic-, and colonic CD compared to HC. 4E-BP1, Eukaryotic translation initiation factor 4E binding protein 1; CASP-8, caspase-8; CCL, C-C motif chemokine; CD40, Tumor necrosis factor receptor superfamily member 5; CD8A, cluster of differentiation 8a; CDCP1, CUB domain-containing protein 1; CXCL, C-XC motif chemokine; EN-RAGE, protein S100-A12; FGF, fibroblast growth factor; FDR, false discovery rate; IL, interleukin; IL-18R1, interleukin-18 receptor 1; IFN-gamma, interferon gamma; MCP, monocyte chemotactic protein; MMP, Matrix metalloproteinase; OPG, osteoprotegerin; OSM, oncostatin M; SLAMF1, signaling lymphocytic activation molecule 1; SIRT2, SIR2-like protein 2; STAMBP, STAM-binding protein; TGF-alpha, transforming growth factor alpha; TNFSF14, tumor necrosis factor ligand superfamily member 14; TRANCE, TNF-related activation-induced cytokine; VEGFA, vascular endothelial growth factor A.

We next tested for differences in individual relative protein levels between UC (*n* = 639), ileal CD (*n* = 275), colonic CD (*n* = 188), ileocolonic CD (*n* = 420). and HC (*n* = 312). Twenty-nine inflammatory-related proteins had significantly higher levels in UC and all CD phenotypes compared to HC, while only fibroblast growth factor (FGF)-19 levels were significantly lower (Figure 1, Supplementary Table 7). FGF-19 remained significantly lower in all CD phenotypes and UC compared to HC, even after adjusting for age, sex, disease duration, and exclusion of patients with previous IBD-related surgery (Supplementary Figures 5 and 6).

In addition, C-X-C motif chemokine (CXCL) 10, C-C motif chemokine (CCL) 11, IL-10, and IL-17A displayed significantly higher relative levels (FDR < 0.05, absolute FC > 1.2) in patients with colonic inflammation (UC, colonic CD, and ileocolonic CD) compared to HC (Figure 1, Supplementary Table 7). No significant differences in these proteins with fold-changes >1.2 were observed in patients with isolated ileal CD compared to HC.

**Figure 2.**
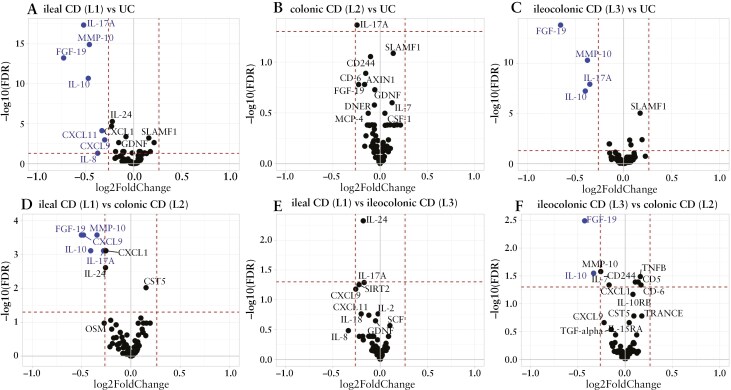
Volcano plots of inflammatory proteins from patients of groupwise comparisons based on fold-change >1.2 (equal to ~0.26 on a log2 scale) and false discovery rate (FDR) < 0.05. (A) ileal Crohn’s disease (CD) versus ulcerative colitis (UC); (B) colonic CD versus UC; (C) ileocolonic CD versus UC; (D) ileal CD versus colonic CD; (E) ileal CD versus ileocolonic CD; (F) ileocolonic CD versus colonic CD. CXCL9, C-X-C motif chemokine ligand 9; CXCL11, C-X-C motif chemokine ligand 11; FGF-19, fibroblast growth factor 19; IL, interleukin; MMP-10, matrix metalloproteinase-10.

Comparisons of individual protein levels revealed a consistent pattern of variation according to disease location in patients with IBD (Figure 2, Supplementary Table 7). Matrix metalloproteinase (MMP)-10, IL-17A, FGF-19, IL-10, and CXCL9 were increased in patients with exclusive colonic inflammation, that is, UC and colonic CD, compared to patients with exclusive ileal inflammation, that is, ileal CD. In addition, levels of FGF-19 and IL-10 were increased in UC and colonic CD compared to ileocolonic CD. Boxplots visualize relative protein levels of the 6 proteins with the lowest *p*-values in the ileal CD versus UC comparison in CD subgroups, UC, IBD-U, and HC (Supplementary Figure 7). Detailed results of these comparisons, including *p*-value, FDR value, and log2 fold-change for each protein, are provided in Supplementary Table 7.

### 3.4. Identifying phenotypes across the IBD spectrum based on protein signatures

**Figure 3.**
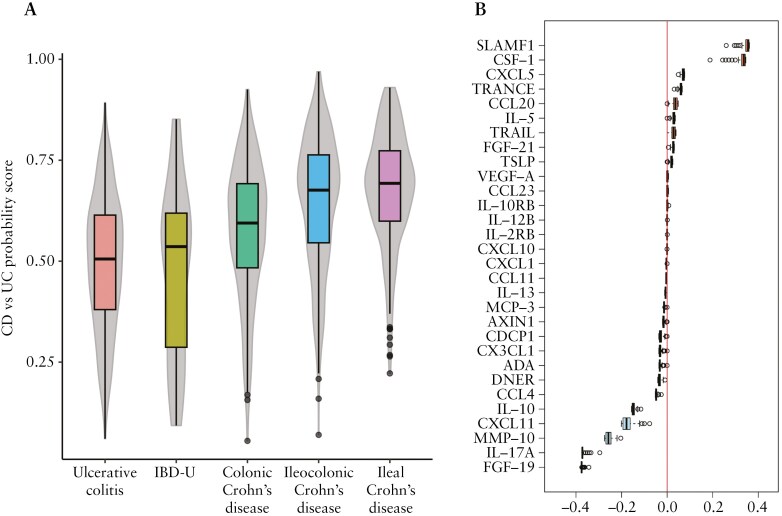
(A) Predicted Crohn’s disease (CD) versus ulcerative colitis (UC) probability estimates from the penalized logistic regression model for UC, IBD unclassified (IBD-U), and phenotypes of CD. A value closer to 0 indicates a higher predicted probability of UC diagnosis, and a value closer to 1 a higher predicted probability of CD diagnosis. (B) Boxplots show coefficients of the UC versus CD models, indicating the importance of the respective proteins in the model. CCL, C-C motif chemokine; CDCP1, CUB domain-containing protein 1; CX3CL1, C-X3-C motif chemokine ligand 1; CXCL, C-X-C motif chemokine; DNER, Delta/Notch Like EGF Repeat Containing; FGF, fibroblast growth factor; IL, interleukin; IL-10RB, IL-10 receptor subunit beta; MCP-3, monocyte-chemotactic protein 3; MMP-10, matrix metalloproteinase 10; SLAMF1, Signaling lymphocytic activation molecule 1; CSF-1, colony stimulating factor 1; TNF, tumor necrosis factor; TRAIL, NF-related apoptosis inducing ligand; TRANCE, TNF-related activation-induced cytokine; TSLP, thymic stromal lymphopoietin; VEGF-A, vascular endothelial growth factor A.

As an initial approach, we employed a protein model distinguishing CD from UC using PLR and RF and performed leave-one-out cross-validation. The CD versus UC logistic regression models yield a probability estimate for each left-out sample, ranging from 0 (indicating a higher predicted probability of a UC diagnosis) to 1 (suggesting a higher predicted probability of a CD diagnosis), thereby classifying patients along the observed CD–UC continuum. To further characterize the spectrum, we examined the CD versus UC estimates across the different CD phenotypes. We, therefore, estimated the median CD versus UC probability scores for each group: UC, IBD-U, colonic CD, ileocolonic CD, and ileal CD (Figure 3A). We observed a spectrum of IBD subgroups, with UC and ileal CD demonstrating the most distinct separation based on the CD versus UC probability score (Supplementary Figure 9). Probability scores significantly differed between colonic CD and UC (*p* < 0.001), ileal CD compared to colonic CD (*p* < 0.001), but not between ileal CD and ileocolonic CD (*p* = 0.07). The most important proteins selected in the CD versus UC model and shaping our observed spectrum included IL-17A, MMP-10, FGF-19, IL-10, signaling lymphocytic activation molecule (SLAMF1), CXCL10, and C-C motif chemokine (CCL)-4 as visualized in boxplots showing the coefficients of the model (Figure 3B). We used the AUC–ROC to explore the performance of the CD versus UC models and achieved an overall AUC of 0.75. While restricting the samples to UC and ileal CD yielded an AUC of 0.81, restricting the samples to UC and colonic samples only yielded an AUC of 0.65. Interestingly, based on the AUC, the UC versus CD scores were slightly, but not significantly (*p* = 0.07) better in segregating colonic IBD from ileal IBD (AUC: 0.78) compared with UC from CD (AUC: 0.75). Based on medical review, experienced physicians reassessed the phenotype of patients with contradictive CD versus UC probability scores to explore the potential clinical impact of using these scores. The rephenotyping of 12 patients with colonic disease (colonic CD and UC, *n* = 6 each) and relatively high estimates, as well as of 6 patients with ileal CD and low estimates, raised doubts about the initial diagnosis in 28% (5 of 18) of these cases. We investigated the correlation between log2-transformed CRP values and FCP with the obtained probability scores to control for the influence of systemic and luminal inflammation. A significant positive correlation was observed for log2CRP, while a negative correlation was found for log2FCP. However, neither correlation was considered clinically relevant, as both were weak (*R*-squared < 0.04) (Supplementary Figure 9). To account for the potential influence of previous and ongoing IBD treatment, we repeated the analyses by excluding SIC IBD samples during model fitting and estimated probability scores of the left-out samples from only treatment-naïve patients. This approach yielded a pattern similar to the pattern observed in the leave-one-out cross-validation approach on the entire dataset (Supplementary Figure 10). Additionally, results from RF and partial least squares analyses demonstrated a pattern consistent with the probability scores obtained using PLR (Supplementary Figures 11A–C and 12A–C).

### 3.5. Evaluating diagnostic potential of inflammatory proteins based on classification models

**Figure 4.**
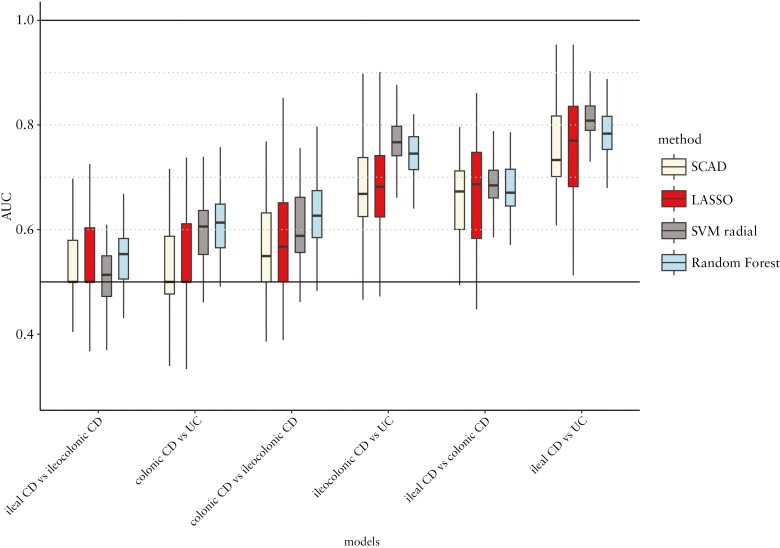
Comparison of the performance of the different models delineating down-sampled subgroups of inflammatory bowel disease (IBD) (each *N* = 188). Performance was evaluated in a repeated 5-fold cross-validation. Area under the receiver-operating characteristic curves (AUCs) of the Random Forest (RF), least absolute shrinkage and selection operator (Lasso)-penalized logistic regression, smoothly clipped absolute deviations (SCAD) combined with ridge-penalized logistic regression, and regularized support vector machine with a radial kernel (SVM radial). SVMs were fitted by selected most important proteins for the model based on variable importance in the inner cross-validation. CD, Crohn’s disease; UC, ulcerative colitis.

Finally, we searched for IBD subgroups based on protein signatures. As a performance metric, we used the AUC since it encapsulates both specificity and sensitivity. Irrespective of the method adopted (least absolute shrinkage and selection operator [Lasso] or SCAD penalized logistic regression, support vector machine, or RF), the models exhibited limited abilities in discriminating between UC and colonic CD, with median AUCs < 0.62 (Figure 4). In contrast, the models separating UC from ileal CD demonstrated a higher performance, with median AUCs > 0.73, and the models differentiating colonic CD from ileal CD showed modest discriminative capacity (median AUCs between 0.67 and 0.69). Median AUCs of CD versus UC models were between 0.67 and 0.74, while median AUCs were between 0.74 and 0.78 of ileal CD versus colonic IBD models (Supplementary Figure 13). These different capabilities of the protein classification models to differentiate between subtypes demonstrate that the location of inflammation appears to exert a more pronounced effect at a systemic level, that is, on serum proteins, compared to the diagnosis alone, and highlights the distinction between ileal-dominant CD and pure colonic CD.

## 4. Discussion

In this comprehensive cross-sectional study of >1800 individuals from multiple European cohorts, we delineated the inflammatory serum protein landscape in IBD across various subtypes. Our results demonstrated a lack of clear segregation in the overall protein profile of CD and UC, as the 2 main subtypes shared several proteins with significantly altered levels compared to controls. Instead, a continuum of IBD phenotypes from ileal CD over colonic CD to UC was identified. Differences in serum protein expression seemed more pronounced between colonic and ileal CD than between colonic CD and UC.

Some previous omics-based studies have suggested that colonic CD represents an intermediate subtype between UC and ileal CD. Based on polygenic risk scores, Cleynen et al.^11^ showed that ileal CD, colonic CD, and UC are entities along a genetic continuum in a large multinational IBD cohort. In alignment with this, transcriptomic profiling of circulating CD4 T-cells has demonstrated marked molecular differences across the IBD spectrum.^10^ In a recent multiomics study of 163 patients with IBD, differences in the patterns of ileal and colonic inflammation were described based on analyses of the fecal metabolome, metaproteome, and microbiota composition.^12^ Our work complements these findings by examining serum protein profiles as an additional informative layer for defining the intricate landscape of IBD and identifying colonic CD as an intermediate between ileal CD and UC.

Our analysis disclosed significantly different levels of specific proteins between subgroups of IBD, among which IL-17A, MMP-10, IL-10, and FGF-19 represented the top proteins when comparing colonic-dominant subtypes IBD (colonic CD or UC) to ileal CD. Also, CXCL11, SLAMF1, and CSF-1 were important for the CD versus UC probability scores in the multivariable PLR model, indicating their role in shaping the IBD spectrum.

Some of the proteins identified in our study have been highlighted in previous research related to IBD phenotypes, primarily in comparisons between CD and UC. Bourgonje et al.^24^ analyzed data from the 1000IBD study, which included 567 patients with CD and 461 with UC, using the same Olink panel. They reported significantly lower relative levels of MMP-10, IL-10, and FGF-19 in quiescent CD compared to quiescent UC. Furthermore, they found differences in FGF-21, IFN-gamma, TNFRSF9, CXCL10, CXCL9, and OPG between CD and UC.^24^ Our study also detected significant differences in FGF-19, MMP-10, IL-10, and CXCL9 when comparing ileal CD and UC, as well as between ileal CD and colonic CD. While we did not observe significant differences in CXCL10 and OPG levels between ileal CD and UC, higher CXCL10 levels were found in all subtypes compared to HC, except for ileal CD. Additionally, we observed higher OPG levels in patients with ileal disease compared to HC. In agreement with our findings, Bourgonje et al. also reported lower levels of FGF-19 in ileal compared to colonic CD. Kalla et al. also compared serum protein levels between CD and UC patients, and among reported significant differences, IL-17A and MMP-10 overlapped with our findings.^25^ Notably, some of the individuals (*n* = 86) included in the study by Kalla et al.^25^ were also part of our dataset. Additionally, a study of 355 healthy first-degree relatives of CD patients found that especially CXCL9 is elevated in first-degree relatives who later in life were diagnosed with CD compared to those who were not diagnosed with the disease during follow-up. The authors also reported elevated CXCL9 levels in first-degree relatives later in life diagnosed with CD with increased intestinal permeability based on the lactulose-mannitol ratio.^26^ In our study, CXCL9 levels were significantly higher in UC and colonic CD compared to ileal CD, although levels were elevated in all subtypes compared to HC.

Beyond protein analyses, other molecular data have linked IL-17A, a key component of the Th17-inflammatory pathway, to IBD phenotypes.^27–29^ Consistent with our findings of higher IL-17A levels in IBD subgroups with colonic inflammation, Bogaert et al. observed pronounced upregulation of IL-17A gene expression in inflamed colonic biopsies, particularly in patients with UC.^30^ Single-cell experiments based on inflamed colonic biopsies have also indicated greater involvement of IL-17 pathways in UC compared to CD.^31^ A previous Phase II/III trial in moderate to severe CD failed to demonstrate a difference between secukinumab, an anti-IL-17A monoclonal antibody, and placebo, but stratified analyses by disease location were not reported.^32^ Therefore, at present, the therapeutic implications of colonic upregulation are contradictory.

We also observed significantly elevated MMP-10 in patients with only colonic inflammation. Metalloproteinases fulfill important functions in extracellular matrix remodeling and wound healing,^33^ yet they are also implicated in tissue breakdown and inflammation. Data from *MMP10* knockout mouse models,^34^ along with previously reported upregulation of MMP-10 in serum during the preclinical phase of UC, suggest that MMP-10 may predominantly exhibit protective properties with preservation of mucosal homeostasis.^35^ Our current findings extend this observation by demonstrating that MMP-10 is also relevant in colonic CD. In contrast, the contribution of MMP-10 to the pathophysiology of ileal inflammation appears to be less significant.

Consistent with previous studies, downregulation of FGF-19 was seen in ileal CD.^18,24^ FGF-19 is expressed in different cell types along the gastrointestinal tract, such as proximal enterocytes.^36^ The prominence of FGF-19 in the signatures that discriminated ileal-dominant CD from colonic subtypes points to low FGF-19 level as a marker of ileal inflammation and suggests impaired reabsorption of bile acids in patients with ileal CD.^37^ Previous studies indicate that the microbiome in ileal CD may be shaped by bile acid malabsorption, whereas neutrophil activity seems more pronounced in colonic disease.^12^

The observed significant influence of disease location on the inflammatory serum proteome may have implications for designing therapeutic randomized trials, clinical practice, and future translational research in IBD. Given the unique composition and physiological characteristics of the ileal and colonic mucosa, it is plausible that the etiological pathways and their associated inflammatory profiles in the blood may differ, as earlier suggested.^9,10,12^ This heterogeneity may subsequently affect the efficacy of a targeted therapy, which could be contingent upon the specific molecular pathways implicated in inflammation. Consequently, the therapeutic efficacy in ileal-dominant versus colonic-dominant disease may vary due to different underlying mechanisms. Interestingly, the efficacy of different targeted therapies, such as adalimumab, infliximab, and vedolizumab, in CD has been reported to differ depending on the location of inflammation, with lower rates of endoscopic healing in ileal disease.^38–40^ In the VISIBLE 2 trial, the effect of vedolizumab was superior to placebo in patients with colonic and ileocolonic CD, but not in those with ileal disease.^41^ Moreover, a lower therapeutic efficacy in ileal CD was also reported in a recent meta-analysis of ustekinumab, vedolizumab, and anti-TNF trials.^42^ On the contrary, exclusive enteral nutrition has been reported to be more effective in ileal and ileocolonic CD compared with exclusive colonic CD^43^ and future anti-fibrotic drugs may be more relevant in the ileal-dominant subtypes.^44^ As elucidated in our study, the differential protein expression across disease locations strengthens the notion that CD should be divided into an ileal-predominant and a colonic-predominant disease.^6^

There is currently very little information on molecular signatures defining IBD subphenotypes. Our study provides an initial glimpse into the set of circulating proteins that are differentially expressed between ileal CD and UC. While our analysis included a large number of patients across multiple cohorts, it was confined to a panel of 86 inflammatory serum proteins. Even though we only observed a weak correlation between the probability scores and CRP, it is possible that proteins not included in our analyses could be more discriminative of CD and UC-specific inflammation or could highlight other subgroups of patients as suggested by Weiser et al.^45^ We combined data from multiple cohorts collected and generated between 1997 and 2021. While adjustments for batch effects were made, these adjustments may have introduced or masked signals in the data, particularly due to unbalanced group sizes within the batches, which is a limitation of our study. However, the similarity in CD versus UC scores observed when analyzing only treatment-naïve patients, compared to the entire dataset, supports the validity of our results and suggests that our findings are robust and are not dependent on IBD treatments. By combining data from multiple cohorts analyzed in different runs, we aimed to identify general patterns related to IBD subtypes ensuring the generalizability and robustness of these results. Furthermore, our findings represent systemic rather than mucosal signatures, and future research should extend to tissue-based analyses to get additional insights. The absence of genetic data is a significant limitation of our study. Including additional layers of omics data could provide deeper insights into disease pathogenesis and enable conclusions about genetic influences.^46^ Eight of the proteins that differed between IBD subtypes and HC in our study have previously been associated with IBD susceptibility based on genomic data (CCL11, CCL20, CD40, CXCL1, CXCL5, IL-10, MCP-4, TRANCE), of which 4 proteins were common for all IBD subtypes versus HC in our analysis (CCL20, CD40, CXCL1, CXCL5).^47^ This overlap and previously reported protein quantitative trait loci (pQTLs) analyses of proteins, such as CXCL5 and CXCL1, suggest a genetic basis for driving changes in levels of some of the identified proteins.^24,47^ Future research should further explore the genetic influence on protein levels within the IBD spectrum.

In summary, our results highlight the importance of redefining the classification of CD into ileal-predominant and colonic-predominant disease. In alignment with previous literature, the findings presented here support the concept of a need for distinct ileal versus colonic-specific therapies in IBD. Stratification of patients according to inflamed segments (ie, UC, colonic CD, and ileal CD) could also offer new insights into IBD pathophysiology.

## Supplementary Material

jjae169_suppl_Supplementary_Materials

jjae169_suppl_Supplementary_Table_S4

jjae169_suppl_Supplementary_Table_S7

## Data Availability

The data underlying this article cannot be shared publicly due to the privacy of the included patients and healthy control participants. The data will be shared on reasonable request to the corresponding author.

## Previous Presentation

Part of the material has been presented at the 19^th^ ECCO Congress 2024 in Stockholm (https://doi.org/10.1093/ecco-jcc/jjad212.0435).

## References

[1] Dolinger M , Torres J, Vermeire S. Crohn’s disease. Lancet 2024;403:1177–91. 38437854 10.1016/S0140-6736(23)02586-2

[2] Le Berre C , Honap S, Peyrin-Biroulet L. Ulcerative colitis. Lancet 2023;402:571–84. 37573077 10.1016/S0140-6736(23)00966-2

[3] Maaser C , Sturm A, Vavricka SR, et al.; European Crohn’s and Colitis Organisation [ECCO] and the European Society of Gastrointestinal and Abdominal Radiology [ESGAR]. ECCO-ESGAR guideline for diagnostic assessment in IBD Part 1: initial diagnosis, monitoring of known IBD, detection of complications. J Crohns Colitis 2019;13:144–64. 30137275 10.1093/ecco-jcc/jjy113

[4] Louis E , Collard A, Oger AF, Degroote E, Aboul Nasr El Yafi F, Belaiche J. Behaviour of Crohn’s disease according to the Vienna classification: changing pattern over the course of the disease. Gut 2001;49:777. 11709511 10.1136/gut.49.6.777 PMC1728556

[5] Silverberg MS , Satsangi J, Ahmad T, et al. Toward an integrated clinical, molecular and serological classification of inflammatory bowel disease: report of a working party of the 2005 Montreal World Congress of Gastroenterology. Can J Gastroenterol 2005;19:5A–36A. 10.1155/2005/269076 16151544

[6] Verstockt B , Bressler B, Martinez-Lozano H, McGovern D, Silverberg MS. Time to revisit disease classification in inflammatory bowel disease: is the current classification of inflammatory bowel disease good enough for optimal clinical management? Gastroenterology 2022;162:1370–82. 34995534 10.1053/j.gastro.2021.12.246

[7] Sandborn WJ , Vermeire S, Peyrin-Biroulet L, et al. Etrasimod as induction and maintenance therapy for ulcerative colitis (ELEVATE): two randomised, double-blind, placebo-controlled, phase 3 studies. Lancet 2023;401:1159–71. 36871574 10.1016/S0140-6736(23)00061-2

[8] Ben-Horin S , Novack L, Mao R, et al. Efficacy of biologic drugs in short-duration versus long-duration inflammatory bowel disease: a systematic review and an individual-patient data meta-analysis of randomized controlled trials. Gastroenterology 2022;162:482–94. 34757139 10.1053/j.gastro.2021.10.037

[9] Atreya R , Siegmund B. Location is important: differentiation between ileal and colonic Crohn’s disease. Nat Rev Gastroenterol Hepatol 2021;18:544–58. 33712743 10.1038/s41575-021-00424-6

[10] Verstockt B , Verstockt S, Cremer J, et al. Distinct transcriptional signatures in purified circulating immune cells drive heterogeneity in disease location in IBD. BMJ Open Gastroenterol 2023;10:e001003. 10.1136/bmjgast-2022-001003 PMC9906185 36746519

[11] Cleynen I , Boucher G, Jostins L, et al.; International Inflammatory Bowel Disease Genetics Consortium. Inherited determinants of Crohn’s disease and ulcerative colitis phenotypes: a genetic association study. Lancet 2016;387:156–67. 26490195 10.1016/S0140-6736(15)00465-1 PMC4714968

[12] Gonzalez CG , Mills RH, Zhu Q, et al. Location-specific signatures of Crohn’s disease at a multi-omics scale. Microbiome 2022;10:133. 35999575 10.1186/s40168-022-01331-x PMC9400277

[13] Assarsson E , Lundberg M, Holmquist G, et al. Homogenous 96-Plex PEA immunoassay exhibiting high sensitivity, specificity, and excellent scalability. PLoS One 2014;9:e95192. 24755770 10.1371/journal.pone.0095192 PMC3995906

[14] Gordon H , Biancone L, Fiorino G, et al. ECCO guidelines on inflammatory bowel disease and malignancies. J Crohns Colitis 2023;17:827–54. 36528797 10.1093/ecco-jcc/jjac187

[15] Olink Proteomics. White Paper. Data Normalization and Standardization. 1096, v2.1, 2022-04-08. https://olink.com/knowledge/documents/. Accessed November 1, 2024.

[16] R Core Team. R: A Language and Environment for Statistical Computing. Vienna: R Foundation for Statistical Computing, 2018. https://www.R-project.org/

[17] Benjamini Y , Hochberg Y. Controlling the false discovery rate: a practical and powerful approach to multiple testing. J R Stat Soc Ser B Stat Methodol 1995;57:289–300.

[18] Andersson E , Bergemalm D, Kruse R, et al. Subphenotypes of inflammatory bowel disease are characterized by specific serum protein profiles. PLoS One 2017;12:e0186142. 28982144 10.1371/journal.pone.0186142 PMC5628935

[19] Fan J , Li R. Variable selection via nonconcave penalized likelihood and its oracle properties. J Am Stat Assoc 2001;96:1348–60.

[20] Breheny P , Huang J. Coordinate descent algorithms for nonconvex penalized regression, with applications to biological feature selection. Ann Appl Stat 2011;5:232–53. 22081779 10.1214/10-AOAS388 PMC3212875

[21] Wright MN , Ziegler A. A fast implementation of random forests for high dimensional data in *C++* and *R*. J Stat Softw 2017;77:1–17.

[22] Friedman J , Hastie T, Tibshirani R. Regularization paths for generalized linear models via coordinate descent. J Stat Softw 2010;33:1–22. 20808728 PMC2929880

[23] Karatzoglou A , Smola A, Hornik K, Zeileis A. kernlab. An S4 package for kernel methods in R. J Stat Softw 2004;11:1–20.

[24] Bourgonje AR , Hu S, Spekhorst LM, et al. The effect of phenotype and genotype on the plasma proteome in patients with inflammatory bowel disease. J Crohns Colitis 2021;15:S036–9. 10.1093/ecco-jcc/jjab157 PMC8919819 34491321

[25] Kalla R , Adams AT, Bergemalm D, et al. Serum proteomic profiling at diagnosis predicts clinical course, and need for intensification of treatment in inflammatory bowel disease. J Crohns Colitis 2021;15:699–708. 33201212 10.1093/ecco-jcc/jjaa230 PMC8095384

[26] Leibovitzh H , Lee S-H, Raygoza Garay JA, et al.; Crohn’s and Colitis Canada (CCC) Genetic, Environmental, Microbial (GEM) Project Research Consortium. Immune response and barrier dysfunction-related proteomic signatures in preclinical phase of Crohn’s disease highlight earliest events of pathogenesis. Gut 2023;72:1462–71. 36788016 10.1136/gutjnl-2022-328421

[27] Öhman L , Dahlén R, Isaksson S, et al. Serum IL-17A in newly diagnosed treatment-naive patients with ulcerative colitis reflects clinical disease severity and predicts the course of disease:. Inflamm Bowel Dis 2013;19:2433–9. 23966065 10.1097/MIB.0b013e3182a563cb

[28] Brand S. Crohn’s disease: Th1, Th17 or both? The change of a paradigm: new immunological and genetic insights implicate Th17 cells in the pathogenesis of Crohn’s disease. Gut 2009;58:1152–67. 19592695 10.1136/gut.2008.163667

[29] Kim SW , Kim ES, Moon CM, et al. Genetic polymorphisms of IL-23R and IL-17A and novel insights into their associations with inflammatory bowel disease. Gut 2011;60:1527–36. 21672939 10.1136/gut.2011.238477

[30] Bogaert S , Laukens D, Peeters H, et al. Differential mucosal expression of Th17-related genes between the inflamed colon and ileum of patients with inflammatory bowel disease. BMC Immunol 2010;11:61. 21144017 10.1186/1471-2172-11-61 PMC3016394

[31] Verstockt S , Ver Donck F, Verstockt B, et al. P827 Up-regulation of IL17-related pathways in affected colon from ulcerative colitis compared with Crohn’s disease. J Crohns Colitis 2019;13:S537–8.

[32] Hueber W , Sands BE, Lewitzky S, et al.; Secukinumab in Crohn's Disease Study Group. Secukinumab, a human anti-IL-17A monoclonal antibody, for moderate to severe Crohn’s disease: unexpected results of a randomised, double-blind placebo-controlled trial. Gut 2012;61:1693–700. 22595313 10.1136/gutjnl-2011-301668 PMC4902107

[33] Murphy G , Nagase H. Progress in matrix metalloproteinase research. Mol Aspects Med 2008;29:290–308. 18619669 10.1016/j.mam.2008.05.002 PMC2810947

[34] Koller FL , Dozier EA, Nam KT, et al. Lack of MMP10 exacerbates experimental colitis and promotes development of inflammation-associated colonic dysplasia. Lab Invest 2012;92:1749–59. 23044923 10.1038/labinvest.2012.141 PMC3510327

[35] Bergemalm D , Andersson E, Hultdin J, et al. Systemic inflammation in preclinical ulcerative colitis. Gastroenterology 2021;161:1526–39.e9. 34298022 10.1053/j.gastro.2021.07.026

[36] Thul PJ , Lindskog C. The human protein atlas: a spatial map of the human proteome. Protein Sci 2018;27:233–44. 28940711 10.1002/pro.3307 PMC5734309

[37] Lenicek M , Duricova D, Komarek V, et al. Bile acid malabsorption in inflammatory bowel disease: assessment by serum markers. Inflamm Bowel Dis 2011;17:1322–7. 21058331 10.1002/ibd.21502

[38] Reinisch W , Colombel J-F, D’Haens G, et al. Characterisation of mucosal healing with adalimumab treatment in patients with moderately to severely active Crohn’s disease: results from the EXTEND trial. J Crohns Colitis 2016;11:425–434. 10.1093/ecco-jcc/jjw178 PMC5881717 27815351

[39] Rivière P , D’Haens G, Peyrin-Biroulet L, et al. Location but not severity of endoscopic lesions influences endoscopic remission rates in Crohn’s disease: a post hoc analysis of TAILORIX. Am J Gastroenterol 2021;116:134–41. 33177349 10.14309/ajg.0000000000000834

[40] Danese S , Sandborn WJ, Colombel J-F, et al. Endoscopic, radiologic, and histologic healing with vedolizumab in patients with active Crohn’s disease. Gastroenterology 2019;157:1007–18.e7. 31279871 10.1053/j.gastro.2019.06.038

[41] Vermeire S , D’Haens G, Baert F, et al. Efficacy and safety of subcutaneous vedolizumab in patients with moderately to severely active Crohn’s disease: results from the VISIBLE 2 randomised trial. J Crohns Colitis 2022;16:27–38. 34402887 10.1093/ecco-jcc/jjab133 PMC8797168

[42] Dulai PS , Singh S, Vande Casteele N, et al. Should we divide Crohn’s disease into ileum-dominant and isolated colonic diseases? Clin Gastroenterol Hepatol 2019;17:2634–43. 31009791 10.1016/j.cgh.2019.04.040 PMC6885453

[43] Xu Y , Guo Z, Cao L, et al. Isolated colonic Crohn’s disease is associated with a reduced response to exclusive enteral nutrition compared to ileal or ileocolonic disease. Clin Nutr 2019;38:1629–35. 30193873 10.1016/j.clnu.2018.08.022

[44] Rieder F , Mukherjee PK, Massey WJ, Wang Y, Fiocchi C. Fibrosis in IBD: from pathogenesis to therapeutic targets. Gut 2024;73:854–66. 38233198 10.1136/gutjnl-2023-329963 PMC10997492

[45] Weiser M , Simon JM, Kochar B, et al. Molecular classification of Crohn’s disease reveals two clinically relevant subtypes. Gut 2018;67:36–42. 27742763 10.1136/gutjnl-2016-312518 PMC5426990

[46] Lloyd-Price J , Arze C, Ananthakrishnan AN, et al.; IBDMDB Investigators. Multi-omics of the gut microbial ecosystem in inflammatory bowel diseases. Nature 2019;569:655–62. 31142855 10.1038/s41586-019-1237-9 PMC6650278

[47] Mirkov MU , Verstockt B, Cleynen I. Genetics of inflammatory bowel disease: beyond NOD2. Lancet Gastroenterol Hepatol 2017;2:224–34. 28404137 10.1016/S2468-1253(16)30111-X

