# Supplementary material for: Characterization of Inflammatory Bowel Disease Heterogeneity Using Serum Proteomics: A Multicenter Study
Source: J Crohns Colitis. 2024 Nov 4;19(5):jjae169. doi: 10.1093/ecco-jcc/jjae169 (PMC12063088; doi:10.1093/ecco-jcc/jjae169)
Supplement: jjae169_suppl_Supplementary_Materials [file jjae169_suppl_supplementary_materials.pdf]

Supplementary File to the manuscript:

Characterization of IBD heterogeneity using serum proteomics: A multicenter study

Authors:

Benita Salomon*[1], Padhmanand Sudhakar*[2,3], Daniel Bergemalm[4], Erik Andersson[4], Olle Grännö[5], Marie Carlson[6], Charlotte R. H. Hedin[7,8], Johan D. Söderholm[9,10], Lena Öhman[11], the BIO IBD consortium, the COLLIBRI consortium, Carl Mårten Lindqvist[1], Robert Kruse[1,12,13], Dirk Repsilber[1], Bram Verstockt[2,14], Séverine Vermeire**[2], Jonas Halfvarson**[4]

* shared first authorship, ** shared last authorship

Affiliations:

1. School of Medical Sciences, Faculty of Medicine and Health, Örebro University, Örebro, Sweden
2. Department of Chronic Diseases and Metabolism, Translational Research Center for Gastrointestinal Disorders (TARGID), KU Leuven, Leuven, Belgium
3. Department of Biotechnology, Kumaraguru College of Technology, Coimbatore, Tamil Nadu, India
4. Department of Gastroenterology, Faculty of Medicine and Health, Örebro University, Örebro, Sweden

5. Department of Laboratory Medicine, Clinical Microbiology, Faculty of Medicine and Health, Örebro University, Örebro, Sweden
6. Department of Medical Sciences, Gastroenterology Research Group, Uppsala University, Uppsala, Sweden
7. Department of Medicine Solna, Karolinska Institute, Stockholm, Sweden
8. Department of Gastroenterology, Dermatovenereology and Rheumatology, Centre for Digestive Health, Karolinska University Hospital, Stockholm, Sweden
9. Department of Surgery, Linköping University, Linköping, Sweden
10. Department of Biomedical and Clinical Sciences, Linköping University, Linköping, Sweden
11. Department of Microbiology and Immunology, Institute of Biomedicine, Sahlgrenska Academy, University of Gothenburg, Sweden
12. Inflammatory Response and Infection Susceptibility Centre, (iRiSC), Faculty of Medicine and Health, Örebro University, Örebro, Sweden
13. Department of Clinical Research Laboratory, Faculty of Medicine and Health, Örebro University, Örebro, Sweden
14. Department of Gastroenterology and Hepatology, KU Leuven, University Hospitals Leuven, Leuven, Belgium

Correspondence:

Corresponding author: Jonas Halfvarson,

Address: Department of Gastroenterology, Faculty of Medicine and Health, Örebro University, SE 70182 Örebro, Sweden

Telephone: +46 19 303000 (switchboard)

Email: jonas.halfvarsson@regionorebrolan.se

Acknowledgement:

The COLLIBRI consortium includes: Ryan C Ungaro[1], Konrad Aden[2], Geert D'Haens[3], Mark S Silverberg[4]

1. Icahn School of Medicine at Mount Sinai, The Dr. Henry D. Janowitz Division of Gastroenterology- Department of Medicine, New York- NY, United States
2. Christian-Albrechts-University and University Hospital Schleswig-Holstein, Institute of Clinical Molecular Biology, Kiel, Germany
3. Amsterdam University Medical Center, Department of Gastroenterology and Hepatology, Amsterdam, The Netherlands
4. Mount Sinai Hospital, Inflammatory Bowel Disease Centre, Toronto- ON, Canada

The BIO IBD consortium includes: Sven Almer[1-2], Francesca Bresso[2], Adam Carstens[3-4], Mauro D’Amato[5-7], Carl Eriksson[8], Henrik Hjortswang[9], Åsa V. Keita[10], Maria Ling Lundström[11], Maria K. Magnusson[12], Jóhann P Hreinsson[13], Hans Strid[2]

1. Department of Medicine Solna, Karolinska Institutet, Stockholm, Sweden
2. Department of Gastroenterology, Dermatovenereology and Rheumatology, Centre for Digestive Health, Karolinska University Hospital, Stockholm, Sweden
3. Department of Internal Medicine, Ersta Hospital, Stockholm, Sweden
4. Department of Medicine Huddinge, Karolinska Institute, Stockholm, Sweden
5. Gastrointestinal Genetics Lab, CIC bioGUNE - BRTA, Derio, Spain
6. Ikerbasque, Basque Foundation for Science, Bilbao, Spain
7. Department of Medicine and Surgery, LUM University, Casamassima, Italy
8. Department of Gastroenterology, Faculty of Medicine and Health, Örebro University, Örebro, Sweden

9. Department of Gastroenterology, County Council of Östergötland, Department of Clinical and Experimental Medicine, Linköping University, Linköping, Sweden
10. Department of Biomedical and Clinical Sciences, Linköping University, Linköping, Sweden
11. Department of Medical Sciences, Gastroenterology Research Group, Uppsala University, Uppsala, Sweden
12. Department of Microbiology and Immunology, Institute of Biomedicine, Sahlgrenska Academy, University of Gothenburg, Sweden
13. Department of Molecular and Clinical Medicine, Sahlgrenska Academy, Göteborg, Sweden

Contents

Supplementary Methods 7
Supplementary Methods 1: R packages 7
Supplementary Methods 2: Samples preparation across studies 8
Supplementary Methods 3: Adjustment for batch effects 9
Supplementary Methods 4: Adjustment of data to control for effects of disease duration, age and sex 10
Supplementary Methods 5: Estimation of CD vs UC diagnostic probability scores using regularized logistic regression. 11
Supplementary Methods 6: Partial least squares analyses 12
Supplementary Methods 7: Performance of diagnostic models to specify IBD subtypes 13
Supplementary Figures 15
Supplementary Figure 1: PCA scores plot highlighting CD-subtypes 15
Supplementary Figure 2: PCA scores plot highlighting UC-subtypes 16
Supplementary Figure 3: PCA loadings 17
Supplementary Figure 4: UMAP 18
Supplementary Figure 5: Comparison to healthy controls using 19
adjusted data 19
Supplementary Figure 6: Group-wise comparisons using adjusted data 21
Supplementary Figure 7: Relative protein levels across IBD subgroups and healthy controls 22
Supplementary Figure 8: Probability scores compared between subgroups. 23
Supplementary Figure 9: CRP, fecal calprotectin, and probability scores 24
Supplementary Figure 10: Swedish inception cohort as validation cohort 25
Supplementary Figure 11: Random Forest derived probability scores 26
Supplementary Figure 12: Partial least square results 27
Supplementary Figure 13: Performance of classification models 29
Supplementary Tables 30
Supplementary Table 1: Cohorts and batches 30
Supplementary Table 2. Year of sample collection and analyses across cohorts 31
Supplementary Table 3. Distribution of inflammatory bowel disease subtypes and healthy controls across batches 32
Supplementary Table 4. Characteristics of patients and controls across cohorts 33

Supplementary Table 5. Comparison of C-reactive protein across across IBD subtypes and healthy controls and cohorts 34
Supplementary Table 6. Comparison of faecal calprotectin across IBD subtypes and healthy controls and cohorts 35
Supplementary table 7. Results of the univariate analyses..................................... 36

Supplementary Methods

Supplementary Methods 1: R packages

Data analyses were performed using the R[1] version 4.0.5. and the packages tidyverse[2], dplyr, purr, rsample, Rlabkey, openxlsx, VennDiagram, ggplot2[3], ggrepel[4], reshape2, uwot[5], caret[6], ncvreg[7], glmnet[8], parallel, multtest[9], kernlab[10], pROC[11], vip[12], FSA[13], mixOmics[14]. R-scripts used for the data analyses are available at: https://git.oru.se/collibri/collibri_2024

Supplementary Methods 2: Samples preparation across studies

Samples were treated with slightly differing protocols across cohorts. For the Swedish Inception cohort (SIC IBD) and the biological cohort (BIO IBD) samples were stored in 6 ml tubes (BD Vacutainer (silica) #367819) and centrifugated at 2400 x g (5 min, room temperature). Samples were aliquoted and stored at -80°C). For the other Örebro cohorts, centrifugation was performed at 2400 x g (7 minutes, at room temperature. Samples aliquoted earlier than year 2005 were first stored at -20°C and later moved to -80°C, while samples aliquoted after 2005 were directly stored at -80°C. For samples from Leuven, SST tubes were used and centrifuged at 1300 g (10 minutes, room temperature). Samples were aliquoted and spanned down at 100 g before they were stored at -80°C.

Supplementary Methods 3: Adjustment for batch effects

Data was obtained from different datasets which explains significant batch-effects visible in the principal component analyses (PCA) and Uniform Manifold Approximation and Projection (UMAP). The batches were of different compositions as only some cohorts included healthy control participants. As part of the decision process of batch correction, we also investigated different batch-effect correction methods, such as rank-based inverse-normal transformation, which was previously used by Folkersen et al.[15], as well as correction based on mean and ComBat.[16] Based on results of principal component analyses (PCA), Uniform Manifold Approximation and Projection (UMAP) and cross-validation of IBD vs. healthy controls models using Random Forest (RF) and smoothly clipped absolute deviation (SCAD) penalized logistic regression we decided to adjust for batch effects based on the median NPX value.

For some batches, data below the limit of detection (LOD) were not available and were replaced values below LOD with the LOD value for all batches. All proteins and samples had <10% missing values. Missing values (n= 268) were imputed with the median value per protein and batch. Samples with missing demographic information (sex, age, disease duration, IBD phenotype) were excluded.

Adjustment for batch effects was performed by subtracting the median NPX value from the NPX values for each protein and in each batch using IBD samples only. Next, the respective median was subtracted from all samples, including the control samples, using the following formula for each protein i (assay) and sample j and each batch:

$$NPX_{i,j\,\text{batch-adjusted}} = NPX_{i,j} - \text{median}\,(NPX_{i,\,\text{IBD-samples}})$$

This method was chosen based on Olink's preprocessing step for the intensity normalization, where the median value for each assay and panel is added to the ready-normalized NPX values as a last step.[17]

Supplementary Methods 4: Adjustment of data to control for effects of disease duration, age and sex

In the main part we have used the full, non-adjusted data set. However, we also performed the univariate analyses using adjusted data (**Supplementary Figures 5 and 6**) to control for effects of sex, age, disease duration and previous surgery. We fitted a linear model using the following equation for each protein i to estimate the effects of age and sex on the respective NPX values across all samples j:

$$NPX_{i,j} = \mu_i + \beta_{i,Age}\, Age_j + \beta_{i,Sex}\, Sex_j + \varepsilon_{i,j}$$

In a next step, the following formula was used to calculate the adjusted NPX value of each sample j (IBD and healthy controls):

$$NPX_{adj1,i,j} = NPX_{i,j} - (\beta_{i,Age}\, Age_j + \beta_{i,Sex}\, Sex_j)$$

We then also adjusted the IBD samples for the effect of disease duration:

$$NPX_{adj,i,j} = \mu_i + \beta_{i,disease\_duration}\, Disease\ duration_j + \varepsilon_{i,j}$$

$$NPX_{adj2,i,j} = NPX_{adj1,i,j} - (\beta_{i,disease\_duration}\, Disease\ duration_j)$$

Finally, we also excluded 296 patients who underwent previous IBD-related surgery or with missing information on surgery (n=245). Thereby, 1010 patients with IBD without previous surgery were included in this part of the analyses.

Supplementary Methods 5: Estimation of CD vs UC diagnostic probability scores using regularized logistic regression.

We employed regularized logistic regression models using relative protein levels to predict the probability of CD vs UC diagnosis (1 for CD and 0 for UC). In the logistic model, the standard logistic function is defined as $f(z)=\frac{1}{1+e^{-z}}$, where z in this case is a linear combination of the relative protein levels.[18,19] We employed regularized logistic regression using a maximum likelihood approach and modelled:

$$z = \beta_0 + \beta_1 x_1 + \beta_2 x_2 + \ldots + \beta_i x_i + \varepsilon$$

Where x_1 to x_i represent the relative serum protein levels and β_1 to β_i the coefficients determined in the fitting of the regularized logistic regression, β_0 representing the intercept and ε the model error.[18,19] Nested cross validation with outer leave-one-out cross-validation was performed. Thereby, we applied the fitted model by using relative protein levels of a left-out sample to calculate an estimate for z, $\hat{z}$. To compute the predicted CD vs UC probability score, we transform $\hat{z}$ to p using the function $p = \frac{1}{1+e^{-\hat{z}}}$.[18,19]

Supplementary Methods 6: Partial least squares analyses

We employed partial least squares (PLS) analysis to identify linear combinations of relative protein estimates that best delineate between UC and CD phenotypes, using the mixOmics package.[14]

Supplementary Methods 7: Performance of diagnostic models to specify IBD subtypes

We compared the performance of the individual models classifying UC and CD subtypes using the Area under the receiver operating characteristic curve (AUC). Four different methods were used in parallel. Ten-times repeated 5-fold cross validation was used, resulting in 50 AUCs being estimated per classification task and method. For each repetition, 188 samples per group were randomly selected to ensure both, class balance of the two subgroups and equal group sizes to fit and evaluate the model for each classification task. For all methods, each fitted model was evaluated for the left-out samples (20%) using AUC estimates from the pROC package[11]. More specifically, we used A) support vector machines with a radial kernel, B) Random Forest, C) Combined L2 and SCAD penalized logistic regression, D) L1 penalized logistic regression. The individual settings for the fitting of the models were chosen as follows:

A) Support vector machines (SVM) were fitted using the packages kernlab[10], caret and vip. A radial kernel ("svmRadial") was selected, and variable selection was performed in an inner cross validation. Variable importance (vip) was estimated using permutations and the AUC as metric. Proteins with an estimated vip > 0 were included in the model. The SVM model using the selected proteins was then fitted and the model performance was then estimated using the left-out samples for each fold.

B) RF models were fitted using the R packages ranger and caret. Hyperparameters, such as minimal node size and number of used proteins per split were optimized in an inner 5-fold cross-validation using kappa[20] as metric. Maximal 500 trees were included in the model.

C) The ncvreg package was used for the SCAD/L2 penalized logistic regression model. Lambda was optimized in an inner cross validation and the value for alpha, determining

L2 (alpha = 0) or SCAD (alpha = 1) contribution to the penalization, was set to 0.1. The parameter k defining the approximate maximum number of proteins allowed in the model was set to 28.

D) For the lasso model, we used the R package glmnet. The parameter alpha, determining contribution of L1 and L2 penalization was set to 1 (only L1 penalization). As for SCAD/L2-penalized logistic regression, we optimized lambda in an inner 5-fold cross validation.

Supplementary Figures

Supplementary Figure 1: PCA scores plot highlighting CD-subtypes

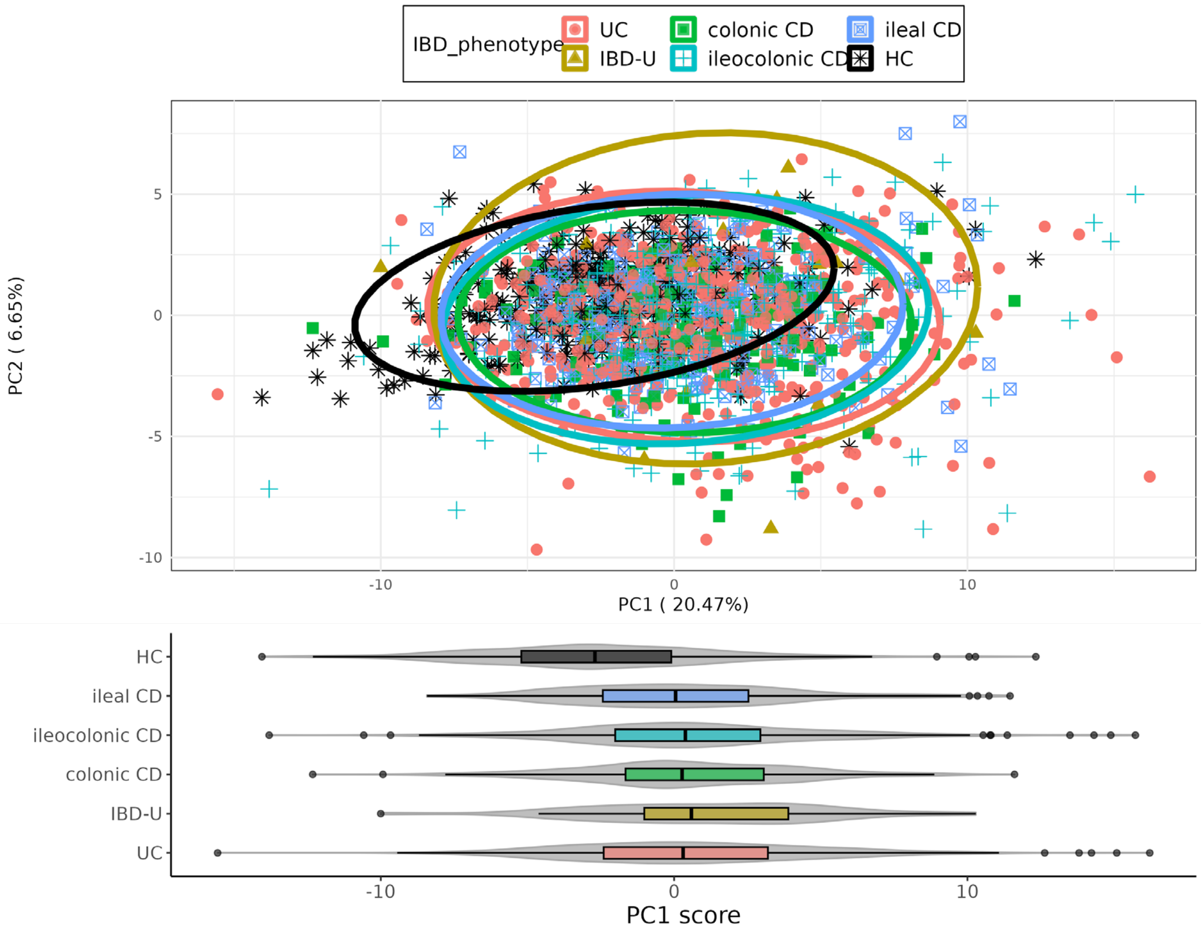

Supplementary Figure 1. Principal component analysis (PCA) scores plot for 1863 samples based on 86 inflammatory proteins showing the degree of discrimination between subgroups of inflammatory bowel disease (IBD). Ulcerative colitis (UC), inflammatory bowel disease-unclassified (IBD-U), colonic Crohn's disease (CD), ileocolonic CD, and ileal CD and healthy controls (HC) are shown in different colours. PC1 scores of HC differed significantly compared with all other groups ($p<0.0001$), while no significant differences were observed among the IBD subgroups based on Kruskal Wallis and Dunn's test.

Supplementary Figure 2: PCA scores plot highlighting UC-subtypes

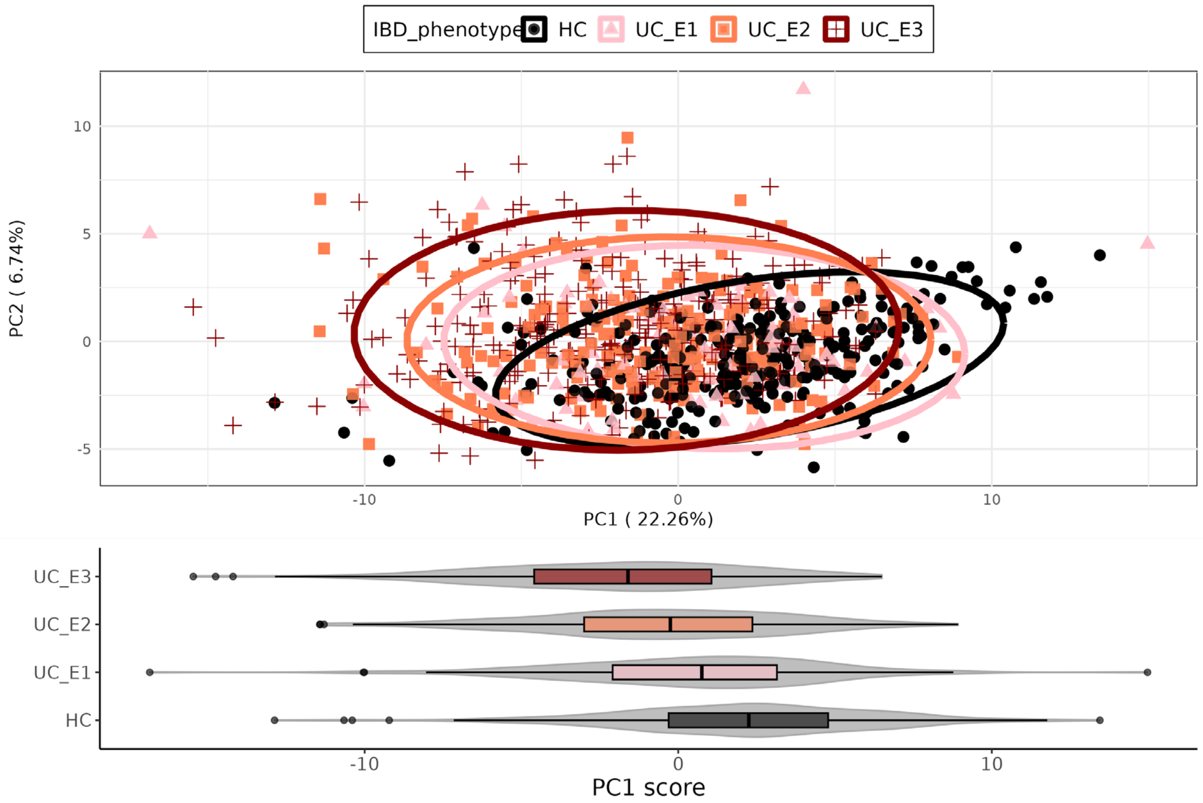

Supplementary Figure 2. Principal component analyses scores plot visualizing CD, IBD-U, UC phenotypes [extensive colitis (E3); left sided colitis (E2); proctitis (E1)] and healthy controls (HC) in different colors. We observed significant differences between the PC1 scores of patients with different UC extents and compared to HC (HC vs E1: p=0.001; E1 vs E2: p<0.0001; E2 vs E3: p=0.0008) based on Kruskal-Wallis and Dunn's test.

Supplementary Figure 3: PCA loadings

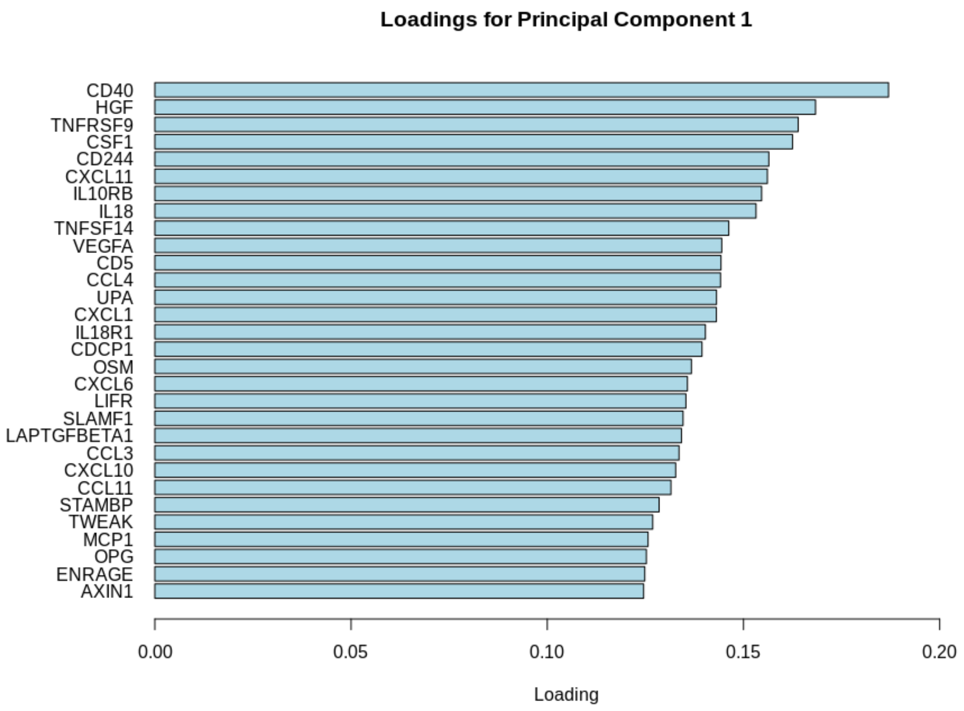

Supplementary Figure 3. The loading plot displays the top 20 proteins with the highest loadings of principal component 1 in the principal component analysis (PCA).

Supplementary Figure 4: UMAP

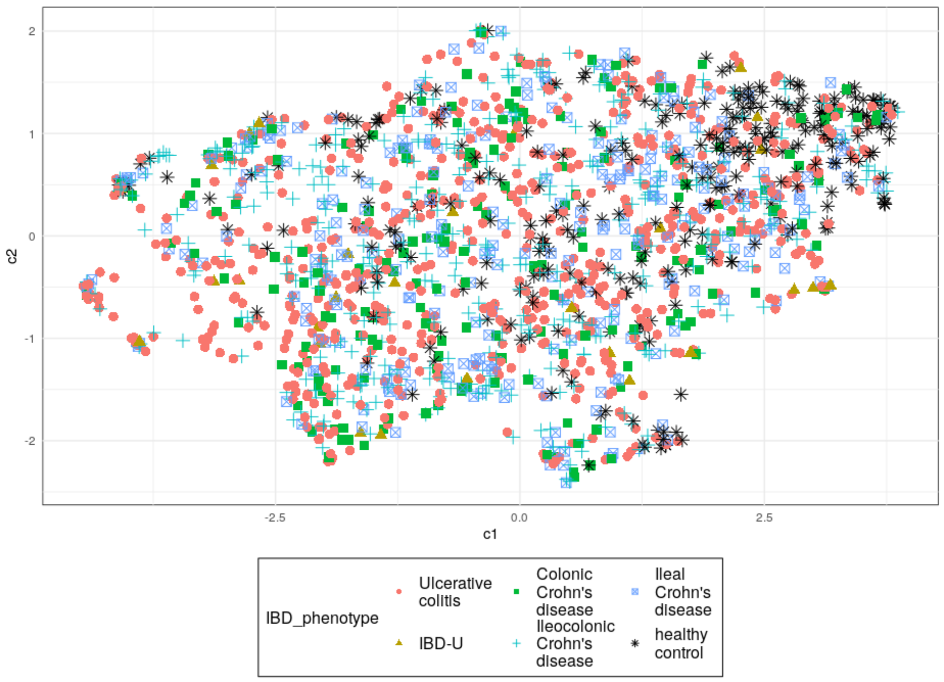

Supplementary Figure 4. Uniform Manifold Approximation and Projection (UMAP) was performed and subgroups of IBD as well as healthy controls are visualized in different colors as an alternative to PCA. IBD subgroups have overlapping clusters. Also, clusters of healthy control and IBD subgroups are overlapping, however, the cluster of healthy controls displays higher density compared to IBD.

Supplementary Figure 5: Comparison to healthy controls using adjusted data

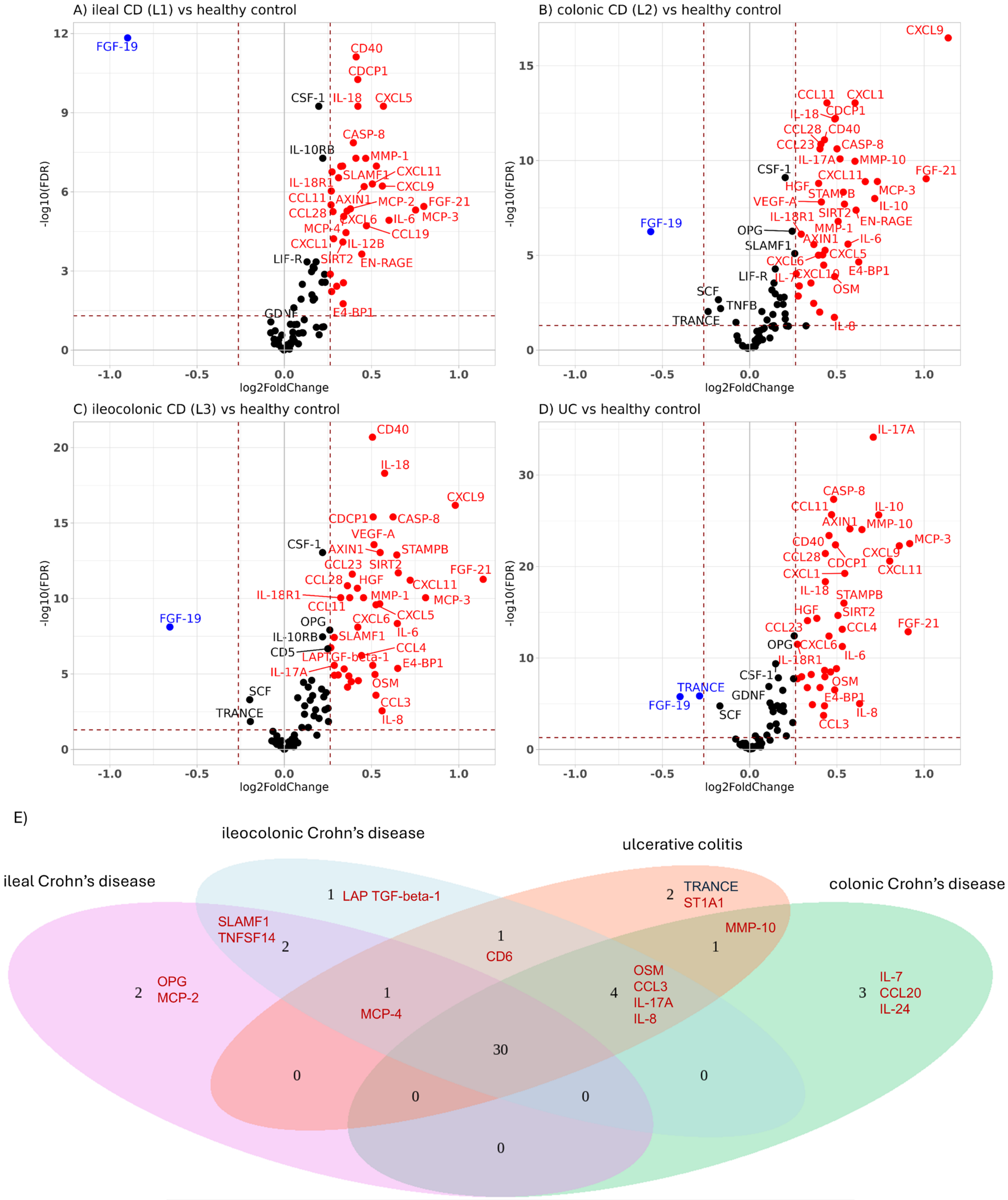

Common	VEGF-A	CXCL11	CCL4	IL-18R1	CXCL23	SIRT2	FGF-19	MMP-1
proteins	MCP-3	AXIN1	IL-18	CXCL5	CXCL6	CCL28	MCP-2	CCL11
across	CDCP1	CXCL9	FGF-21	HGF	CXCL10	EN-RAGE	CASP8	
all groups:	IL-6	CXCL1	CCL19	IL-10	4E-BP1	CD40	STAMBP	

Supplementary Figure 5. Protein data of patients with ileal Crohn's disease (CD; n=139), colonic CD (n=146), ileocolonic CD (n=209), ulcerative colitis (UC; n=491) and healthy controls (HC; n=238). Protein data were adjusted for estimated effects of age, sex and disease duration. Only patients without previous IBD-related surgery were included in this part of the analyses. A-D) Volcano plots show proteins with higher or lower relative levels in CD-subtypes and UC compared to HC. E) Venn Diagram showing the overlap of proteins higher or lower in the CD subtypes and UC. Analyses were performed using the sex-, age- disease duration adjusted data and exclusion of individuals with previous surgery.

Supplementary Figure 6: Group-wise comparisons using adjusted data

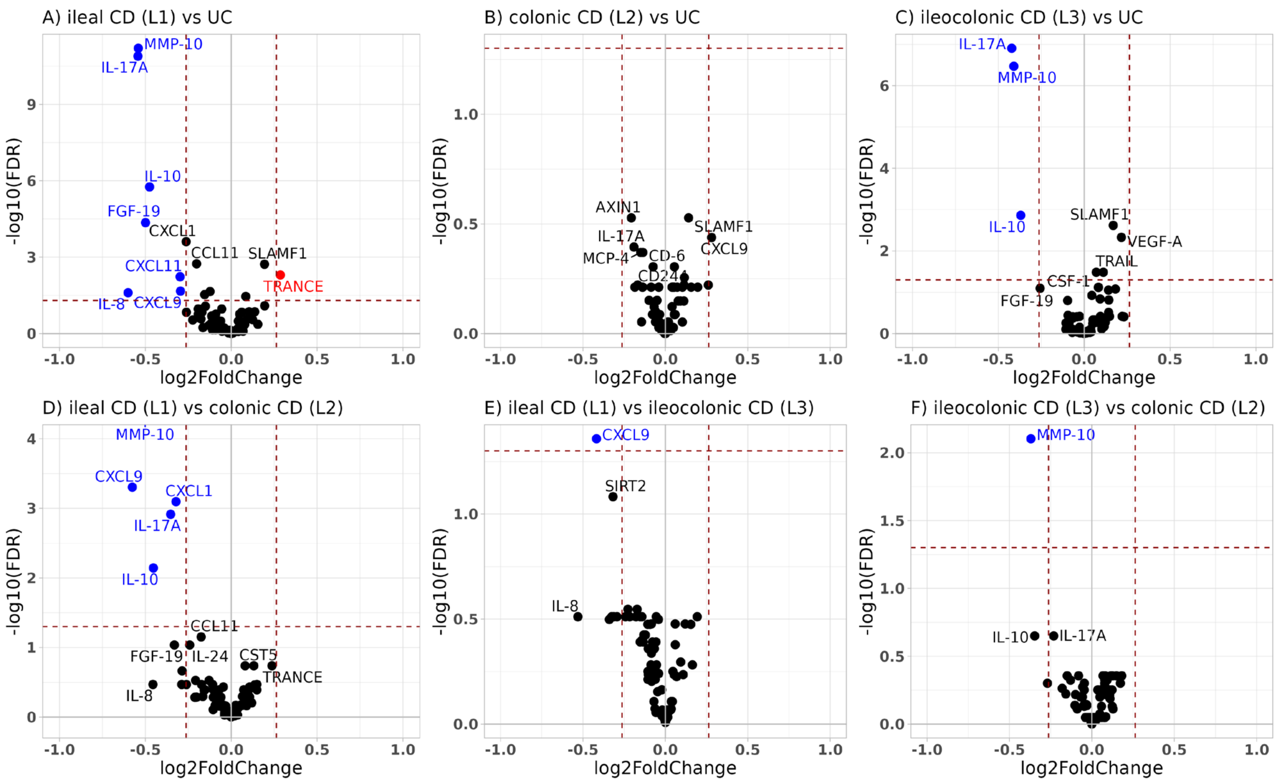

Supplementary Figure 6. Volcano plots showing proteins significantly different in Crohn's disease (CD)-subtypes and ulcerative colitis (UC) in pair-wise comparison using data adjusted for effects of age, sex and disease duration and excluding individuals with previous IBD-related surgery. **A)** ileal CD vs UC **B)** colonic CD vs UC **C)** ileocolonic CD vs UC **D)** ileal CD vs colonic CD **E)** ileal CD vs ileocolonic CD **F)** ileocolonic CD vs colonic CD. Similar proteins were found here, using the adjusted data compared to non-adjusted data (Figure 3). However, we observed no significant, but still a tendency difference (FDR < 0.1) of FGF-19 in ileal CD vs colonic CD and ileocolonic CD compared to UC.

Supplementary Figure 7: Relative protein levels across IBD subgroups and healthy controls

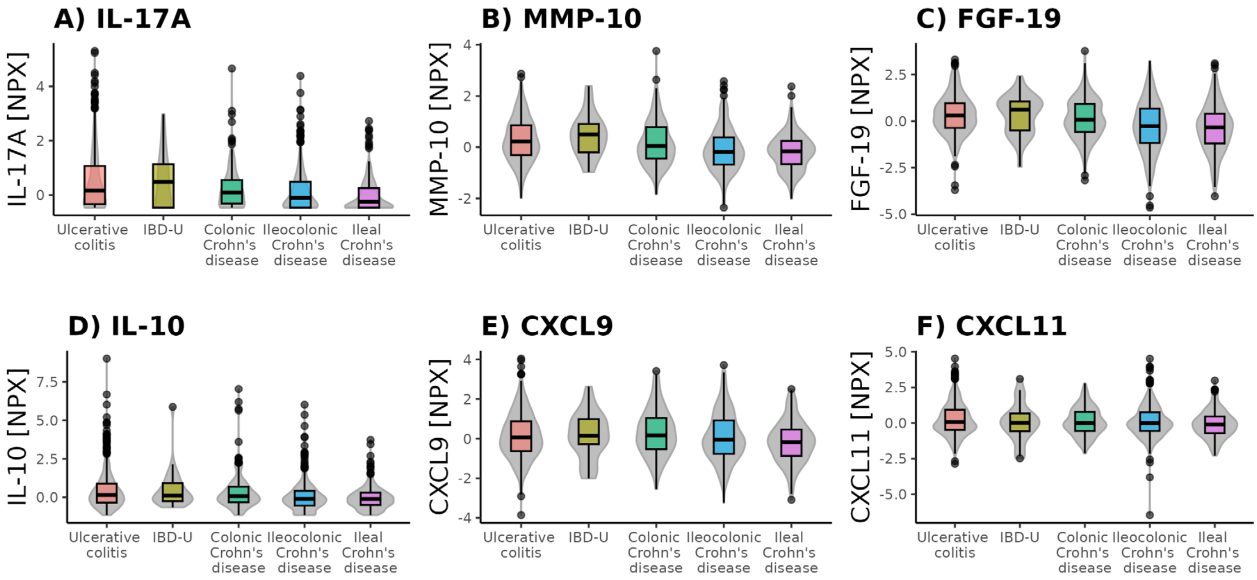

Supplementary Figure 7. Boxplots of proteins with significantly different levels in pair-wise comparison of ulcerative colitis (UC) and Crohn's disease (CD) phenotypes. (A) IL-17A (B) MMP-10 (C) FGF-19 (D) IL-10 (E) CXCL9 (F) CXCL11.

Supplementary Figure 8: Probability scores compared between subgroups.

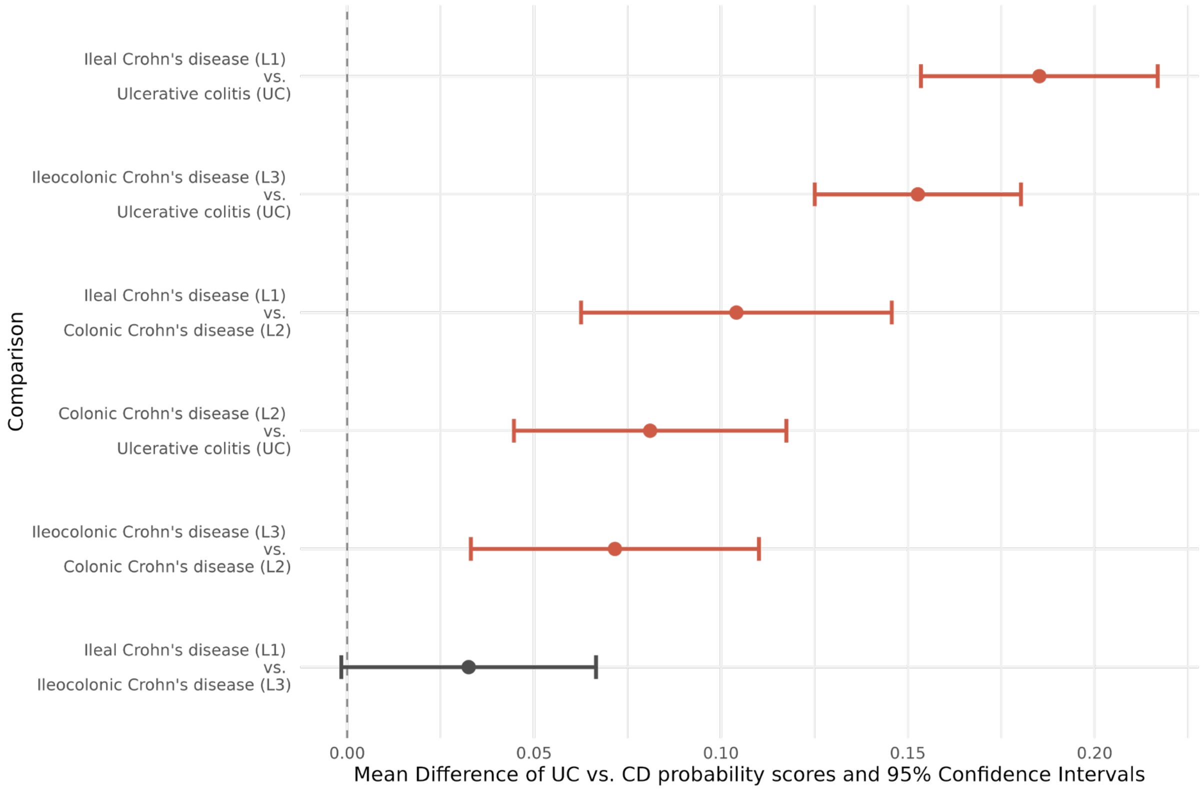

Supplementary Figure 8. Probability scores yielded by penalized logistic regression models segregating CD and UC. Scores of each CD-subgroup and UC were analyzed in a pair-wise comparison. Significant differences were found between all subtypes and UC except for ileal CD vs ileocolonic CD. We observed significant differences between all phenotypes except for ileal CD vs ileocolonic CD. The most pronounced difference was between UC and ileal CD. The difference between colonic CD and UC was marginally, albeit not significantly, less than that between ileal CD and colonic CD.

Supplementary Figure 9: CRP, fecal calprotectin, and probability scores

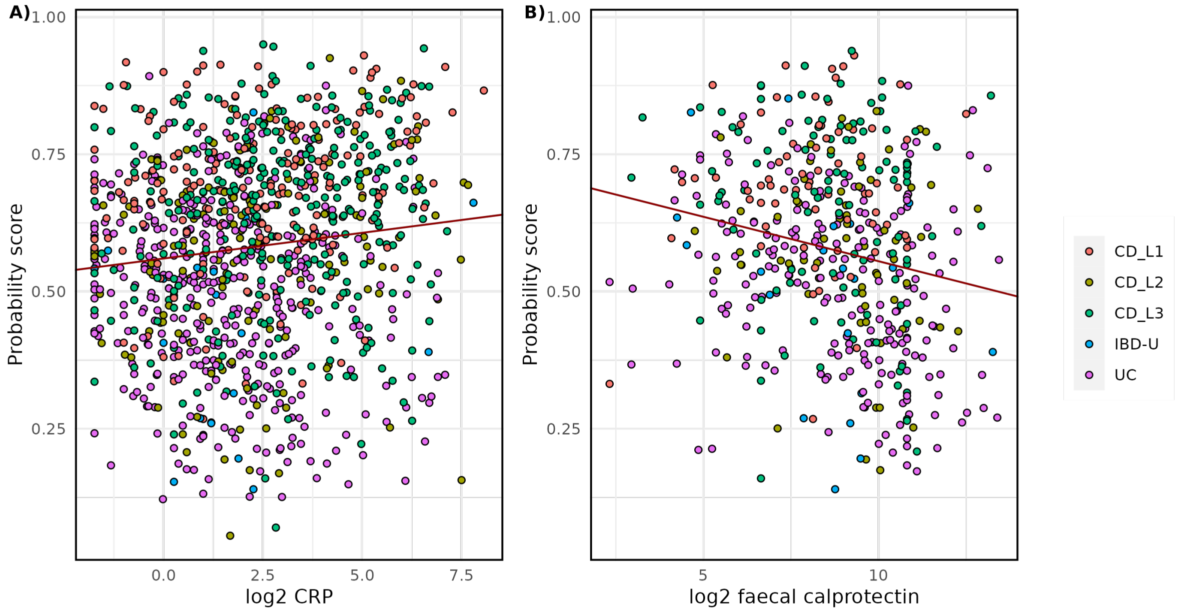

Supplementary Figure 9. A) Correlation of Crohn's disease (CD) vs ulcerative colitis (UC) probability scores and log2 C-reactive protein (CRP) with a subset of patients with available CRP measures (n=1082). The correlation was significant ($p < 0.001$, r=0.12, Rsquared=0.01). **B)** Correlation of CD vs UC probability scores and log2-faecal calprotectin in a subset of patients (n=477) due to missingness of fecal calprotectin measurements. We observed a significant negative correlation ($p < 0.001$, r= -0.20, Rsquared= 0.04). Ileal Crohn's disease, CD L1; colonic Crohn's disease, CD L2; ileocolonic Crohn's disease, CD L3; Inflammatory bowel disease unclassified, IBD-U; ulcerative colitis, UC.

Supplementary Figure 10: Swedish inception cohort as validation cohort

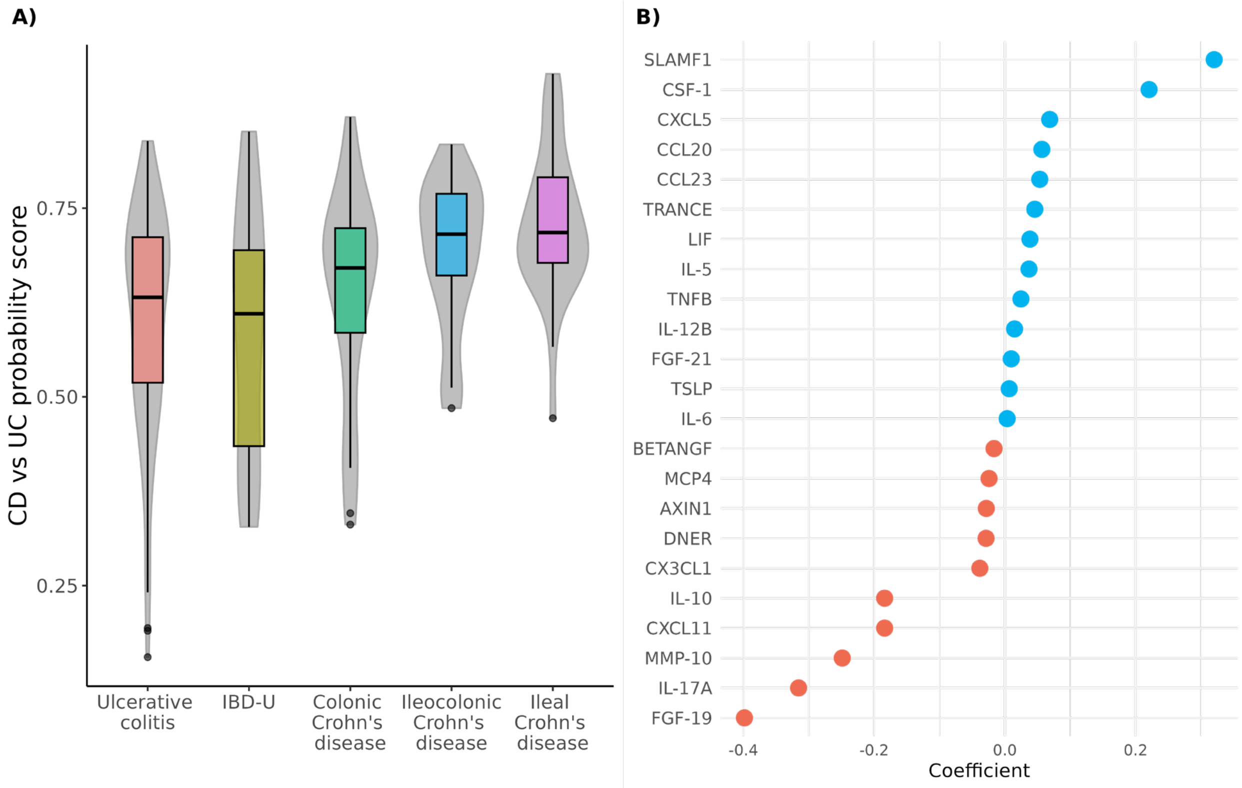

Supplementary Figure 10. A) To account for the potential influence of previous and ongoing IBD treatments, samples from the Swedish Inception Cohort (SIC IBD) were excluded during model fitting. The fitted penalized logistic regression model was then used to estimate Crohn's disease (CD) vs ulcerative colitis (UC) probability scores of the left-out samples from only treatment-naïve patients in the SIC IBD (UC n=149; IBD-U n=21; colonic CD n=31; ileocolonic CD n=21; ileal CD n=32). **B)** Coefficients of the UC vs CD model, indicating the importance of the respective proteins in the model.

Supplementary Figure 11: Random Forest derived probability scores

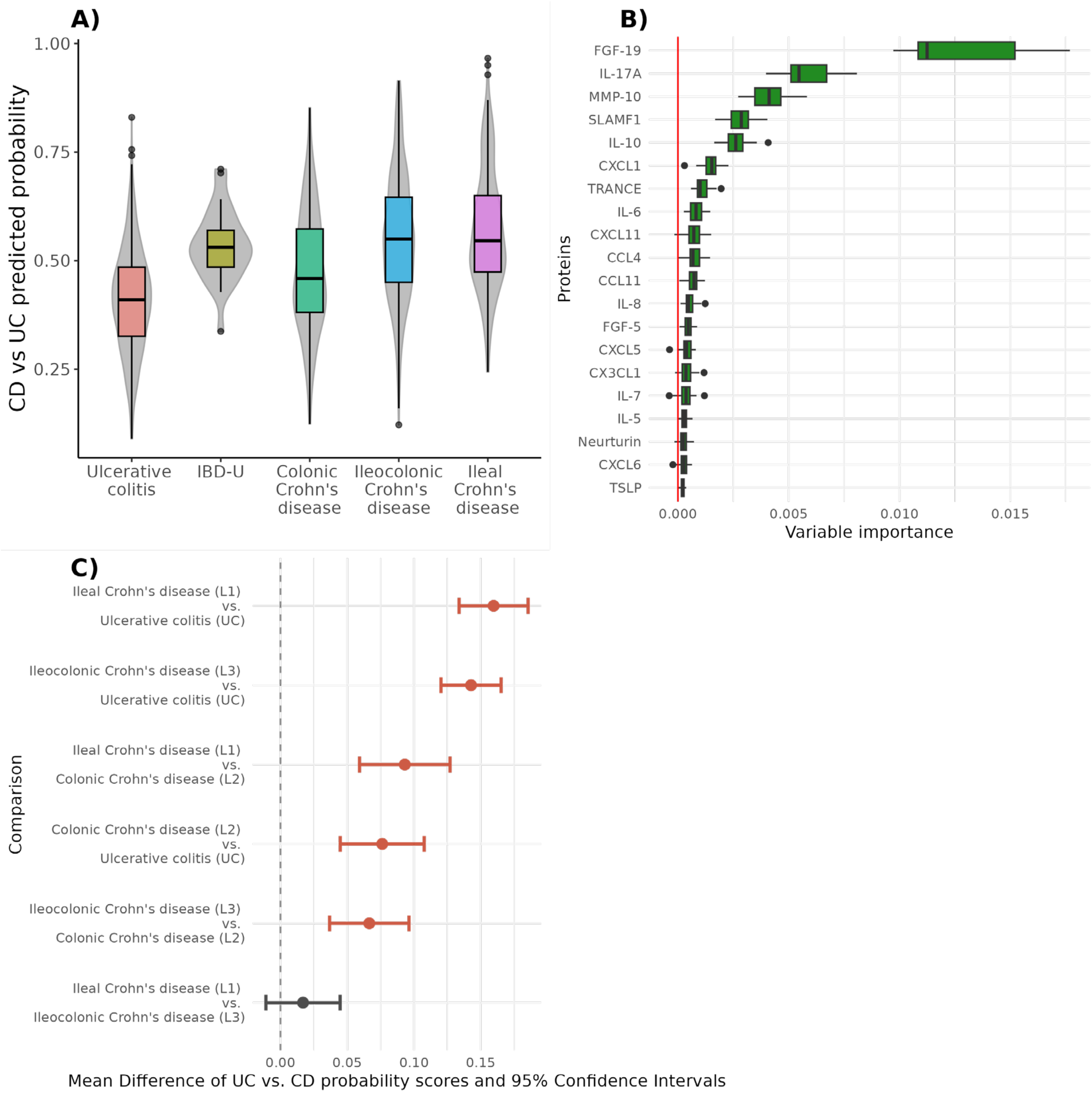

Supplementary Figure 11. Random forest models segregating Crohn's disease (CD) and ulcerative colitis (UC) were used to estimate CD vs UC probability scores. **A)** Probability scores per CD-subtype, IBD-U and UC. **B**) Pairwise comparison of scores. **C)** Variable importance of the top 20 proteins in the models.

Supplementary Figure 12: Partial least square results

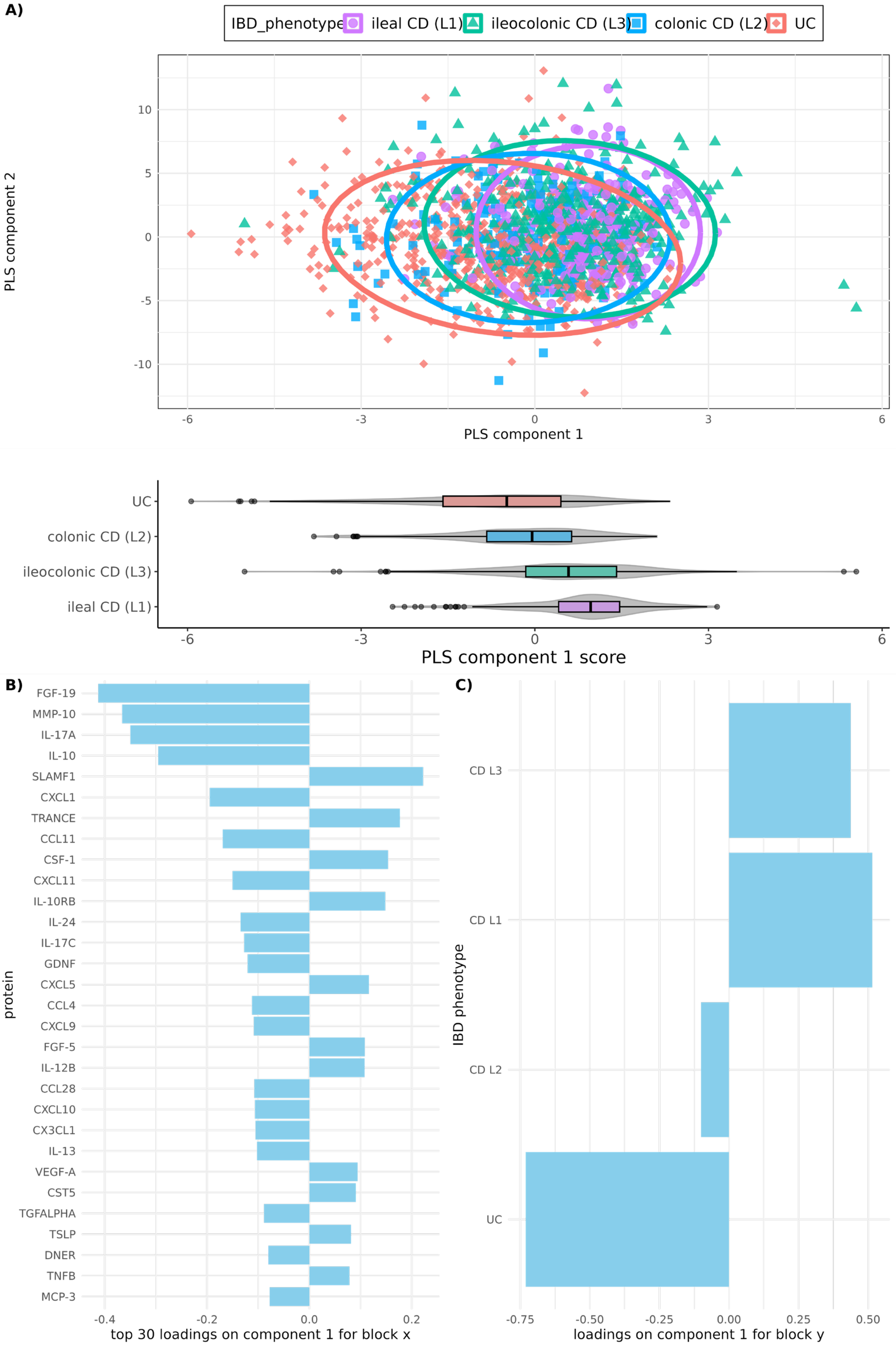

Supplementary Figure 12. We performed partial least square analyses to further address the discriminative potential of serum protein profiles. While some overlap persisted among groups, the spatial separation was most pronounced between ileal CD and UC, and intermediate between colonic CD and ileocolonic CD. Proteins FGF19, IL-17A, SLAMF1, TNF-related activation-induced cytokine (TRANCE) and Macrophage colony-stimulating factor 1 (CSF-1) displayed the highest absolute loadings. **A)** Scores plot of the partial least square analyses and boxplots visualizing the partial least square component 1 scores of the different IBD subtypes. **B-C)** Loading plots of the partial least square analysis. In **B)** only top 30 absolute loadings are displayed.

Supplementary Figure 13: Performance of classification models

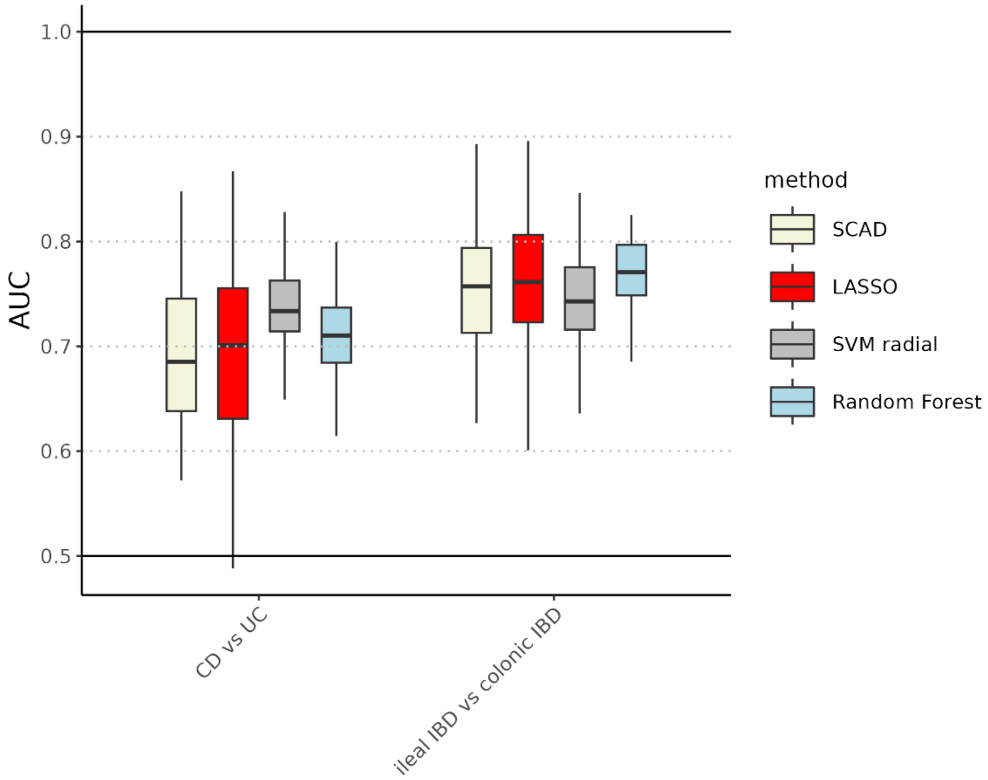

Supplementary Figure 13. Performance of classification model based on the Area under the receiver operating characteristic curve (AUC). The performance of CD vs UC models and ileal IBD vs colonic IBD (UC and colonic CD) was investigated based on different classification models (Random Forest, blue; Support vector machines with radial kernel, grey; L1-penalized logistic regression, red; Smoothly clipped absolute deviation and L2-penalized logistic regression, beige) and in a five-fold cross validation.

Supplementary Tables

Supplementary Table 1: Cohorts and batches

Within this project existing protein data were included and are listed below. For remaining batches adjustment for batch-effects was performed as explained in **Supplementary Methods 4**.

Supplementary Table 1. List of Included cohorts and overview of batches

Main cohort	Batches	Centre
**Swedish Inception cohort (SIC IBD)**	**SIC IBD 1**<br>**SIC IBD 2**<br>**SIC IBD 3**	Two centers<br>Five centers<br>Six centers<br>(Swedish IBD centers)
**Cohort of patients starting biological treatment (BIO IBD)**	**BIO IBD 1**<br>**BIO IBD 2**	Six Swedish IBD centers
**Örebro cohort 1**	**Örebro 1.1**<br><br>**Örebro 1.2**	Örebro University Hospital, Sweden
**Örebro cohort 2**	**Örebro 2**	Örebro University Hospital, Sweden
**Leuven***	**Leuven**	University Hospitals Leuven

*Protein analyses of samples collected at the University Hospital Leuven were performed in three runs, but the results had been bridged into one dataset before the conduction of the current study. Therefore, the dataset from Leuven was treated as one batch.

Supplementary Table 2. Year of sample collection and analyses across cohorts

	Years of sample collection	Years of sample analyses	Version of the inflammation panel	Previous publications
**SIC IBD**	2011-2019	2021	v.3012<br>v.3022	[21]
**BIO IBD**	2015-2019	2018 and 2020	v.3004<br>v. 3022	
**Örebro 1**	2005-2012	2014	-	[22]
**Örebro 2**	2002-2014	2017	v.3003	
**Leuven**	1997-2015	2017-2019	v.3003<br>v.3004<br>v.3011	

Supplementary Table 3. Distribution of inflammatory bowel disease subtypes and healthy controls across batches

	Ileal CD (L1)	Colonic CD (L2)	Ileocolonic CD (L3)	UC	IBD-U	HC
**SIC IBD 1, n (%)**	18 (15.8)	13 (11.4)	12 (10.5)	67 (58.8)	4 (3.5)	0
**SIC IBD 2, n (%)**	11 (8.3)	16 (12)	6 (4.5)	52 (39.1)	0	48 (36.1)
**SIC IBD 3, n (%)**	4 (5.5)	4 (5.5)	6 (8.2)	41 (56.2)	18 (24.7)	0
**BIO IBD 1, n (%)**	8 (11.8)	10 (14.7)	12 (17.6)	38 (55.9)	0	0
**BIO IBD 2, n (%)**	30 (16.9)	45 (25.4)	33 (18.6)	65 (36.7)	4 (2.3)	0
**Örebro 1.1, n (%)**	17 (10.8)	14 (8.9)	21 (13.4)	54 (34.4)	0	51 (32.5)
**Örebro 1.2, n (%)**	5 (5.7)	13 (14.9)	9 (10.3)	30 (34.5)	0	30 (34.5)
**Örebro 2, n (%)**	23 (11.4)	21 (10.4)	17 (8.4)	41 (20.3)	0	100 (49.5)
**Leuven, n (%)**	159 (18.7)	52 (6.1)	304 (35.7)	251 (29.5)	3 (0.4)	83 (9.7)

Inflammatory bowel disease, IBD; Crohn's disease, CD; ulcerative colitis, UC; IBD unclassified, IBD-U; healthy controls, HC; Swedish Inception Cohort, SIC IBD; biological cohort, BIO IBD

Supplementary Table 4. Characteristics of patients and controls across cohorts

Detailed information about characteristics of patients and controls across cohorts is provided as a separate Excel file with multiple sheets.

Supplementary Table 5. Comparison of C-reactive protein across across IBD subtypes and healthy controls and cohorts

		Ileal CD (n=207)	Ileocolonic CD (n=300)	Colonic CD (n=146)	IBD-U (n=22)	UC (n=407)	HC (n=203)	Test (p-value)
**CRP [mg/L]**	**Overall (n=1285)**	4.70 (1.45- 14.65)	7.25 (2.70-24.53)	4.10 (1.60 - 17.68)	1.35 (0.30-2.60)	2.70 (1.15-7.65)	0.33 (0.30- 0.77)	<0.001
	**SIC IBD (n=294)**	6.05 (3.35-17.00)	5.45 (3.35-32.75)	7.50 (1.85-34.00)	2.60 (1.83-4.13)	2.60 (0.89-7.30)	0.63 (0.33-1.10)	<0.001
	**BIO IBD (n=218)**	2.25 (0.74- 6.10)	4.30 (1.90-8.20)	3.40 (1.07-5.85)	3.70 (2.00-4.25)	2.60 (0.85-6.45)	-	0.39
	**Örebro 1 (n=0)**	-	-	-	-	-	-	-
	**Örebro 2 (n=202)**	1.50 (0.72-2.30)	1.60 (0.67-4.10)	2.60 (1.60- 3.30)	-	2.20 (0.94- 3.70)	0.88 (0.36 -1.50)	<0.001
	**Leuven (n=571)**	7.55 (2.00-18.23)	12.20 (3.50- 29.85)	13.90 (4.45-33.20)	1.00 (1.00-1.00)	3.45 (1.70-11.68)	1.00 (0.30-2.05)	<0.001

C-reactive protein, CRP; Crohn’s disease, CD; IBD unclassified, IBD-U; ulcerative colitis, UC; healthy control, HC; Swedish Inception Cohort, SIC IBD; Biological cohort BIO IBD

Supplementary Table 6. Comparison of fecal calprotectin across IBD subtypes and healthy controls and cohorts

		Ileal CD (n=71)	Ileocolonic CD (n=106)	Colonic CD (n=64)	IBD-U (n=20)	UC (n=216)	HC (n=216)	
**FCP [μg/g]**	**Overall (n=517)**	298 (127-627)	488 (179 -1381)	716 (263-1178)	471 (159-654)	579 (137-1645)	10 (5-24)	<0.001
	**SIC IBD (n=247)**	394 (246-538)	469 (130-980)	717 (323-1397)	471 (187-9826)	415 (113-1360)	10 (5-215)	<0.001
	**BIO IBD (n=152)**	227 (5-552)	485 (208-1160)	733 (208-1090)	372 (198-545)	755 (263-1690)	-	0.06
	**Örebro 1 (n=0)**	-	-	-	-	-	-	
	**Örebro 2 (n=0)**	-	-	-	-	-	-	
	**Leuven (n=118)**	286 (118-818)	542 (193-1529)	155 (100-210)	-	832 (255-1800)	-	0.07

Fecal calprotectin, FCP; Crohn's disease, CD; IBD unclassified, IBD-U; ulcerative colitis, UC; healthy control, HC; Swedish Inception Cohort, SIC IBD; Biological cohort BIO IBD

Supplementary table 7. Results of the univariate analyses

Results of the univariate analyses are provided as a separate Excel file including multiple sheets.

References

1 R Core Team. R: A language and environment for statistical computing. R Foundation for Statistical Computing, Vienna, Austria. 2018. https://www.R-project.org/.

2 Wickham H, Averick M, Bryan J, et al. Welcome to the Tidyverse. *J Open Source Softw* 2019; **4**: 1686.

3 Wickham H, Averick M, Bryan J, et al. Welcome to the Tidyverse. *J Open Source Softw* 2019; **4**: 1686

4 Slowikowski K. ggrepel: Automatically Position Non-Overlapping Text Labels with ‘ggplot2’. R package version 0.9.3. 2023. https://CRAN.R-project.org/package=ggrepel.

5 Melville J. uwot: The Uniform Manifold Approximation and Projection (UMAP) Method for Dimensionality Reduction. R package version 0.1.16. 2023. https://CRAN.R-project.org/package=uwot.

6 Kuhn M. Building Predictive Models in *R* Using the caret Package. *J Stat Softw* 2008; **28**.

7 Breheny P, Huang J. Coordinate descent algorithms for nonconvex penalized regression, with applications to biological feature selection. *Ann Appl Stat* 2011; **5**: 232–53.

8 Friedman J, Hastie T, Tibshirani R. Regularization Paths for Generalized Linear Models via Coordinate Descent. *J Stat Softw* 2010; **33**.

9 Pollard KS, Dudoit S, van der Laan MJ. Multiple Testing Procedures: R multtest Package and Applications to Genomics, in Bioinformatics and Computational Biology Solutions Using R and Bioconductor. *Springer Stat Biol Health Ser* 2005. 251–72.

10 Karatzoglou A, Smola A, Hornik K, Zeileis A. kernlab - An *S4* Package for Kernel Methods in *R*. *J Stat Softw* 2004; **11**.

11 Robin X, Turck N, Hainard A, et al. “pROC: an open-source package for R and S+ to analyze and compare ROC curves.” *BMC Bioinformatics* 2011, **12**: 77.

12 Greenwell B M, Boehmke B C. Variable Importance Plots—An Introduction to the vip Package. *R J* 2020; **12**: 343.

13 Ogle DH, Doll JC, Wheeler AP, Dinno A. FSA: Simple Fisheries Stock Assessment Methods. R package version 0.9.5. 2023. https://CRAN.R-project.org/package=FSA.

14 Rohart F, Gautier B, Singh A, Lê Cao K-A. mixOmics: An R package for ‘omics feature selection and multiple data integration. *PLOS Comput Biol* 2017; **13**: e1005752.

15 Folkersen L, Gustafsson S, Wang Q, et al. Genomic and drug target evaluation of 90 cardiovascular proteins in 30,931 individuals. *Nat Metab* 2020; **2**: 1135–48.

16 Leek JT, Johnson WE, Parker HS, Jaffe AE, Storey JD. The sva package for removing batch effects and other unwanted variation in high-throughput experiments. *Bioinformatics* 2012; **28**: 882–3.

17 Olink Proteomics. White Paper. Data normalization and standardization. 1096, v2.1, 2022-04-08. https://olink.com/knowledge/documents/.

18 Peng CY, Lee KL, Ingersoll GM. An introduction to logistic regression analysis and reporting. *The journal of educational research.* 2002; **96**:3-14.

19 Altman DG. *Practical statistics for medical research*. Boca Raton, Fla: Chapman & Hall/CRC, 1999.

20 Warrens MJ. Cohen's kappa is a weighted average. *Stat Methodol* 2011; **8**: 473–84.

21 Bazov I, Kruse R, Bergemalm D, et al. P154 A novel serum protein signature as biomarker for Inflammatory Bowel Disease: A diagnostic performance and prediction modelling study using data from two independent inception cohorts. *J Crohns Colitis* 2023; **17**: i314–5.

22 Andersson E, Bergemalm D, Kruse R, et al. Subphenotypes of inflammatory bowel disease are characterized by specific serum protein profiles. *PLOS ONE* 2017; **12**: e0186142.
